# Systems biology of the modified branched Entner-Doudoroff pathway in *Sulfolobus solfataricus*

**DOI:** 10.1371/journal.pone.0180331

**Published:** 2017-07-10

**Authors:** Ana Sofia Figueiredo, Theresa Kouril, Dominik Esser, Patrick Haferkamp, Patricia Wieloch, Dietmar Schomburg, Peter Ruoff, Bettina Siebers, Jörg Schaber

**Affiliations:** 1 Institute for Experimental Internal Medicine, Medical Faculty Otto von Guericke University, Magdeburg, Germany; 2 Molecular Enzyme Technology and Biochemistry (MEB), Biofilm Centre, Centre for Water and Environmental Research (CWE), Faculty of Chemistry, University of Duisburg-Essen, Essen, Germany; 3 Bioinformatics & Biochemistry, Technische Universität Braunschweig, Braunschweig, Germany; 4 Center for Organelle Research (CORE), University of Stavanger, Stavanger, Norway; Friedrich-Alexander-Universitat Erlangen-Nurnberg, GERMANY

## Abstract

*Sulfolobus solfataricus* is a thermoacidophilic Archaeon that thrives in terrestrial hot springs (solfatares) with optimal growth at 80°C and pH 2–4. It catabolizes specific carbon sources, such as D-glucose, to pyruvate via the modified Entner-Doudoroff (ED) pathway. This pathway has two parallel branches, the semi-phosphorylative and the non-phosphorylative. However, the strategy of *S*.*solfataricus* to endure in such an extreme environment in terms of robustness and adaptation is not yet completely understood. Here, we present the first dynamic mathematical model of the ED pathway parameterized with quantitative experimental data. These data consist of enzyme activities of the branched pathway at 70°C and 80°C and of metabolomics data at the same temperatures for the wild type and for a metabolic engineered knockout of the semi-phosphorylative branch. We use the validated model to address two questions: 1. Is this system more robust to perturbations at its optimal growth temperature? 2. Is the ED robust to deletion and perturbations? We employed a systems biology approach to answer these questions and to gain further knowledge on the emergent properties of this biological system. Specifically, we applied deterministic and stochastic approaches to study the sensitivity and robustness of the system, respectively. The mathematical model we present here, shows that: 1. Steady state metabolite concentrations of the ED pathway are consistently more robust to stochastic internal perturbations at 80°C than at 70°C; 2. These metabolite concentrations are highly robust when faced with the knockout of either branch. Connected with this observation, these two branches show different properties at the level of metabolite production and flux control. These new results reveal how enzyme kinetics and metabolomics synergizes with mathematical modelling to unveil new systemic properties of the ED pathway in *S*.*solfataricus* in terms of its adaptation and robustness.

## Introduction

*Sulfolobus solfataricus* (*S*. *solfataricus*) belongs to the Archaea, the third domain of life. This extremophile grows optimally in hot (80°C), acidic (pH 2–4) environments where few organisms can survive [1, 2], an adaptation whose underlying mechanisms are not well understood. Interestingly, *S*.*solfataricus* shares some traits with organisms of the other two domains of life: bacteria and eukaryotes. One of these traits is the central carbohydrate metabolism (CCM), which resembles those of bacteria and lower eukaryotes in its complexity. *S*. *solfataricus* catabolizes several carbon sources into pyruvate (Pyr), which is then completely oxidized to CO_2_ via the tricarboxylic acid (TCA) cycle, and reducing equivalents are channeled into the branched aerobic respiration chain for energy production. Archaea utilize modified pathways, however, characterized by many novel enzymes sharing no similarity to their bacterial and eukaryotic counterparts. For example, D-glucose (Glc) conversion to Pyr in *S*. *solfataricus* does not proceed via the typical Embden-Meyerhof-Parnas (EMP) pathway (i.e. glycolysis), but through a modified version of the Entner-Doudoroff (ED) pathway [3] found in some aerobic bacteria [4–9] and fungi [10]. This modified ED pathway uses novel biocatalysts and has two branches—the non-phosphorylative (npED) and semi-phosphorylative (spED) [11–13].

The role of the two branches present in the ED pathway has been investigated in several studies [14, 15]. Since neither ED branch allows for ATP gain by substrate level phosphorylation [11], the hypothesis that the parallel branches exist as ATP sources seems very unlikely. Kouril and colleagues suggest that the spED branch has mainly a gluconeogenic function, whereas the npED branch has mainly a glycolytic function [15]. However, the spED branch is in principle capable of both gluconeogenesis and glycolysis and seems to be nessasary for normal cell functions, whereas the npED branch has only glycolytic function and, therefore, seems to be dispensable for gluconeogenesis. Other studies suggest that these two branches may confer “metabolic thermoadaptation” in (hyper)thermophilic Archaea [12, 16], since the npED bypasses the thermolabile intermediates (triosephosposphates: GAP, DHAP and BPG). In all hyperthermophilic Archaea studied so far the regulation of the glycolytic pathways is excerted at the level of triose phosphate conversion by unique enzymes (i.e. catabolic non-phosphorylating GAPN, gluconeogenetic classical GAPDH and PGK, gluconeogenetic bifunctional fructose-bisphosphate aldolase/phosphatase) (for detailed discussion see [11]).

Here, we characterize the steady-state properties of the branched ED pathway in *S*.*solfataricus* in a quantitative manner. To this end, we design a dynamic mathematical model describing the complete ED pathway of *S*. *solfataricus* based on available knowledge. For the first time, we parameterize large parts of this model with enzyme activities from cell-free (crude) extract measurements, published K_m_ values [13, 17–22], metabolomics data and enzymatic half-life measurements. We validate the model using independent experimental data. Finally, we use the validated model to address two questions:

- *Is the ED pathway more robust to perturbations at its optimal growth temperature compared to lower temperatures?* *and*
- *Is this pathway robust to one branch deletion and to uncertainty at the metabolite steady state level*?

Understanding the robustness of *S*. *solfataricus’* CCM is a cornerstone toward understanding how this organism evolved to thrive in hot, acid conditions. The knowledge gained by studying such organisms offers great potential for biotechnological applications, where robust, easy-to- use thermophiles might serve as cell factories to efficiently and economically produce biofuels or fine chemicals under extreme reactor conditions. [23, 24]. The application of mathematical models can optimize the delivery of such results in terms of time and resources.

## Results

### 1. Model implementation and description

**Fig 1:**
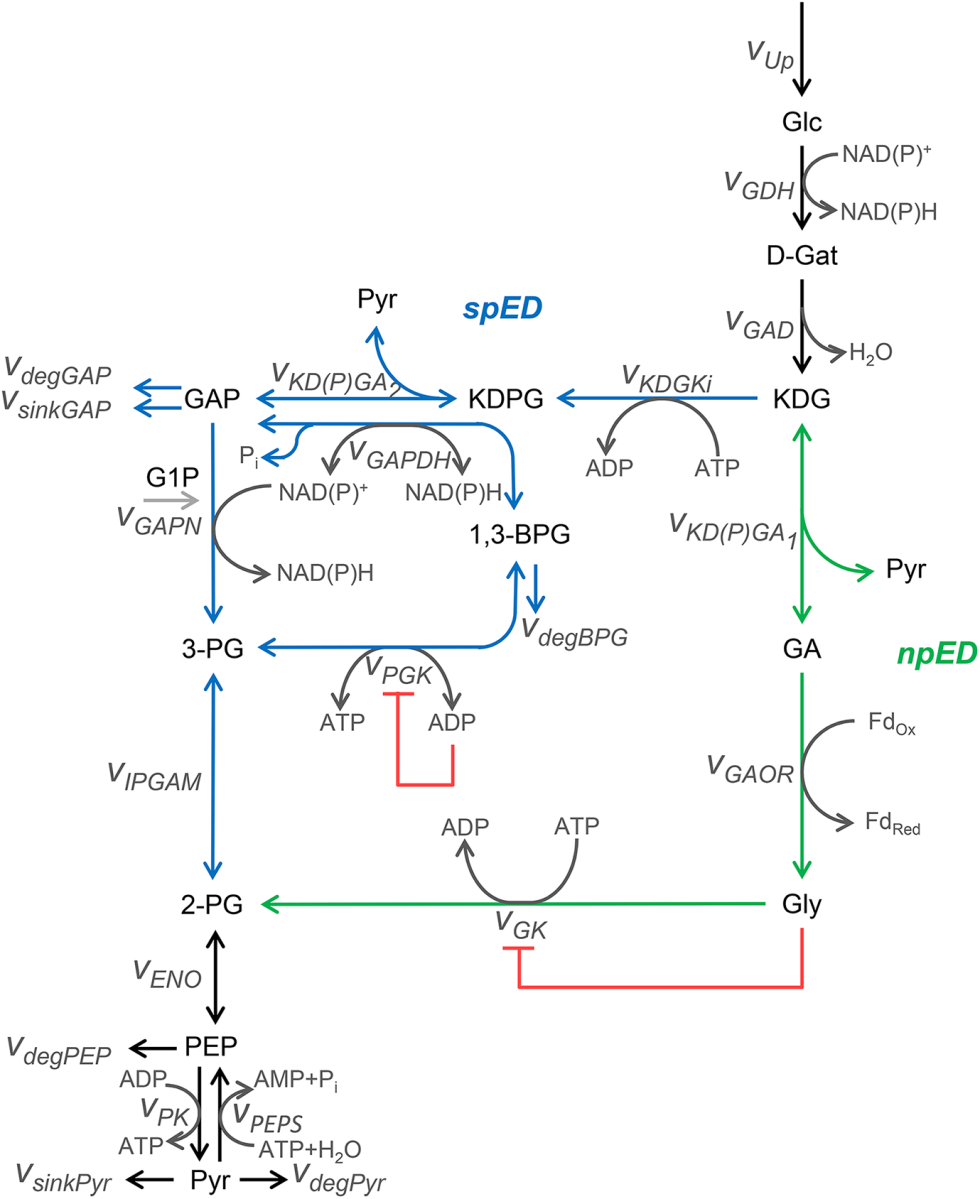
Conceptual model of the Entner-Doudoroff (ED) pathway in *S*.*solfataricus*. This pathway catabolizes D-glucose (Glc) to pyruvate (Pyr) and has two distinct branches: the semi-phosphorylative (spED) in blue and the non-phosphorylative (npED) in green. Both branches incorporate regulatory mechanisms (red lines). Black lines represent the common branches of the network and gray lines represent ADP/ATP phosphorylation/dephosphorylation and NADP/NADPH redox-reactions.

We implemented a kinetic mathematical model using a system of ordinary differential equations (ODEs). Our conceptual model of the ED pathway of *S*. *solfataricus* (Fig 1) combines several published results on partial aspects of the CCM, such as a regulation motif accounting for phosphoglycerate kinase (V_PGK_) inhibition [20] and a feedforward accounting for glycerate kinase (V_GK_) inhibition. All details of the system of ODEs and the respective kinetics of each reaction are described in the S1 and S2 Tables, S1 Dataset.

The ED pathway converts D-glucose (Glc) to pyruvate (Pyr) in *S*.*solfataricus* (for recent review see Bräsen et al. 2014). The glucose uptake rate (V_up_) represents the Glc transport into the cell. Internal Glc converts to D-gluconate (D-Gat) via glucose 1-dehydrogenase (V_GDH_) [17, 25–27]. D-Gat is, in turn, converted to 2-keto-3-deoxygluconate (KDG) via gluconate dehydratase (V_GAD_) [18, 28]. KDG is the metabolite where the semi-phosphorylative (spED) branch and the non-phosphorylative (npED) branch bifurcate [12].

In the spED branch, KDG is first converted to 2-keto-3-deoxy-6 phosphogluconate (KDPG) and then to Glyceraldehyde-3-phosphate (GAP) by the reactions 2-keto-3-deoxy-D-gluconate kinase (V_KDGKi_) and 2-keto-3-deoxy gluconate aldolase (V_KDPG5A2_), respectively [13, 15, 17].

GAP, in turn, can be further transformed to G6P via gluconeogenesis or to Pyr via glycolysis [15, 20]. In glycolysis, GAP is either reversibly converted to 1,3-bisphosphoglycerate (1,3-BPG) by the classical GAPDH reaction (V_GAPDH_) [20, 29] or irreversibly to 3-phosphoglycerate (3-PG) via the unidirectional, non-phosphorylating catabolic enzyme GAPN (V_GAPN_) [21]. GAPN is activated by glucose 1-phosphate (G1P), which is implicitly considered in our mathematical model through the V_max_ and K_m_ values relative to the conversion of GAP to 3-PG. 1,3-BPG, formed by GAPDH, is further converted to 3-PG by the PGK reaction (v_PGK_) [20], which is coupled to ATP formation (substrate level phosphorylation). Detailed enzymatic characterization revealed that v_GAPDH_ and v_PGK_ are gluconeogenic reactions, whereas V_GAPN_ is a glycolytic one. However, in this model, we consider v_GAPDH_ and v_PGK_ as reversible reactions [20]. 3-PG is reversibly converted to 2-PG by the enzyme phosphoglycerate mutase (V_IPGAM_). 2-PG is the metabolite where the spED branch and the npED branch converge.

In the npED branch, KDG is converted to glyceraldehyde (GA) and pyruvate via KD(P)G aldolase (V_KDPGA1_). Glyceraldehyde:ferredoxin oxidoreductase (V_GAOR_) catalyzes the oxidation to glycerate (Gly) using ferredoxin as co-factor [22] and, finally, Gly is transformed to 2-PG via glycerate kinase (V_GK_) [15].

In the lower part of the modified ED pathway (common to both branches), 2-PG is reversibly converted to phosphoenopyruvate (PEP) by enolase (V_ENO_), before pyruvate kinase (V_PK_) produces Pyr and ATP (substrate level phosphorylation). In the anabolic direction, PEP is formed from Pyr via the gluconeogenetic reaction catalyzed by phosphoenolpyruvate synthase (V_PEPS_). Alternatively, Pyr is further oxidized via the TCA cycle and leaves the system through the reaction V_sinkPyr._ For recent review see [11].

Co-factors may work as transductors of signal propagation, in a specific yeast population and under certain well defined conditions that lead to oscillations [30]. In our model, we deal with steady states. Co-factor ratios ATP/ADP and NAD(P)^+^/NAD(P)H are held constant in our model, as previously done in *S*.*solfataricus* [15], as long as we don’t understand how these co-factors are regulated. However, in order to prove our concept we released this constraint and allowed for 25% variation (decrease and increase of ATP, ADP and NAD(P)H^+^ concentration, S1 Fig). These variations had no significant impact at the steady state level of the overall metabolites of the modified ED pathway.

Details for the kinetic model including reaction kinetics, catalyzing enzymes and parameters in the S1 and S2 Datasets. S3 and S4 Tables.

We included reactions for the degradation of the thermolabile intermediates GAP, 1,3-BPG and PEP, based on available metabolite half-life measurements [20, 31]. We also considered sink reactions in those metabolites connecting to other pathways not considered explicitly in this study. Specifically, we provided GAP and Pyr with sink-reactions (V_sinkGAP_ and V_sinkPyr_, respectively). These metabolites connect to and are further used in gluconeogenesis to glycogen and the TCA cycle to CO_2_, respectively.

Generally, biological organisms possess control mechanisms that assure essential physiological activities, despite fluctuations in a determined range of temperatures. This evolutionary trait allows the dynamic adaptation of organisms when faced to temperature changes in a certain range. Specifically, *S*.*solfataricus* grows optimally at around 80°C, but can survive in a wide temperature range. We computed the influence of temperature on the system by applying the Arrhenius equation to individual rate constants (Materials and Methods, Eq (9–11)). Based on these results, we conclude that the enzyme activities account for the system's behavior when temperature is changed from 70°C to 80°C.

## 2. Model parameterization

**Fig 2:**
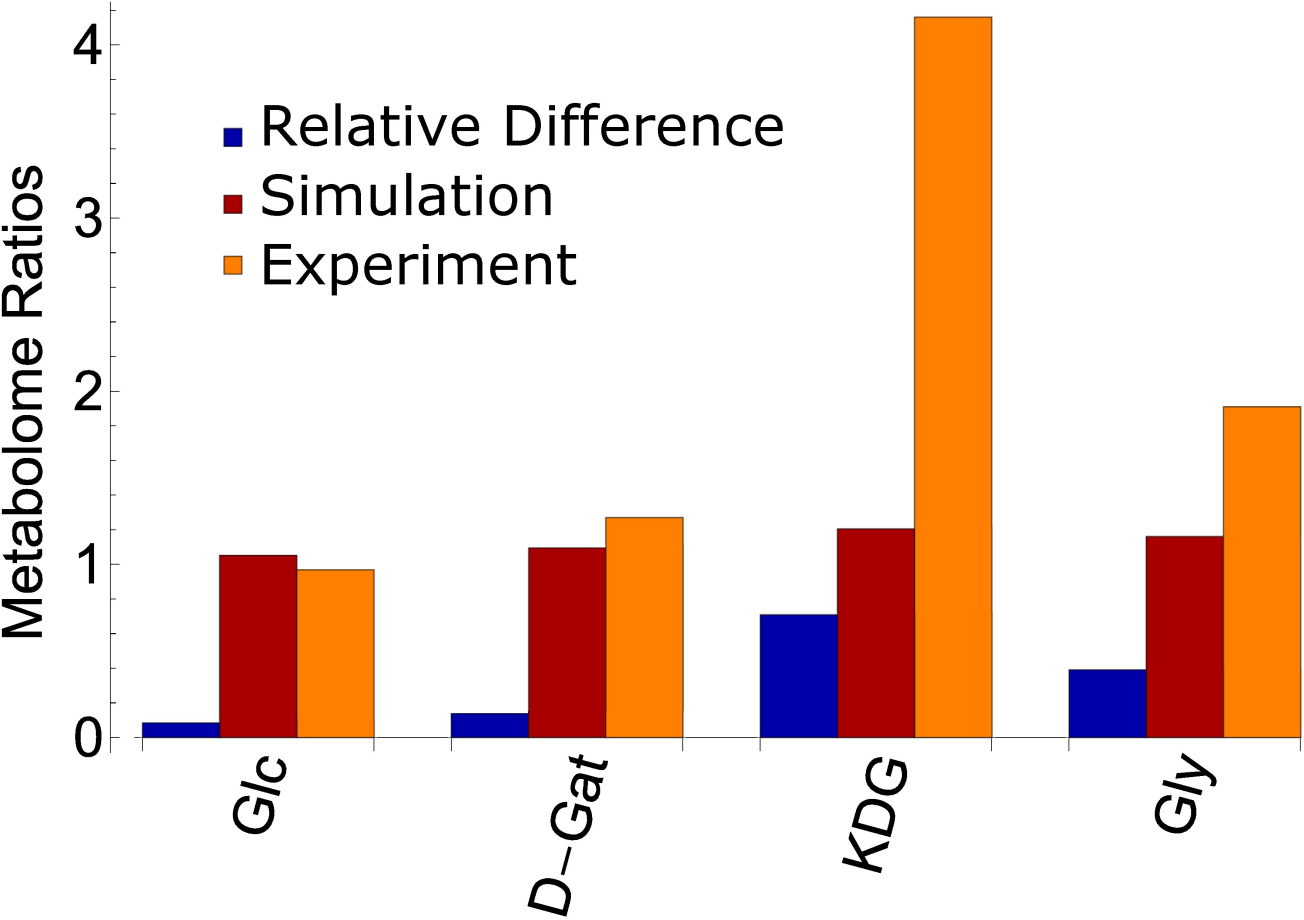
Metabolome ratios for Glc, D-Gat, KDG, GA and Gly. Metabolome ratios calculated with Eq 2. Comparison between the metabolomics experiments (metabolites Glc, D-Gat, KDG and Gly) and simulated metabolite concentrations. Relative difference between simulated and experimental levels in blue; simulated calculations in red; experimental values in orange.

**Parameter estimation.** Specific enzyme activities of 11 out of 25 reactions could not be measured due to low enzyme activities (i.e. technical limitations). Moreover, sink reactions also had to be estimated. To estimate the free enzyme activities $V_{max}^{free}$ and sink rates $V_{sink}^{free}$, we adopted the following strategy:

1. *Assume the validity of the Van’t Hoff rule to recover the steady states at 80°C using the 70°C V_max_ values (Eq 1)*.
  $${SS}^{80{}^{\circ}C}(V_{max,80}^{meas},V_{max,80}^{free},V_{half,80}^{meas},V_{sink,80}^{free})\approx {SS}_{sim}^{80{}^{\circ}C}({2V}_{max,70}^{meas},{2V}_{max,70}^{free},V_{half,80}^{meas},V_{sink,80}^{free})$$ (1)
  Where V_max,T_ indicates V_max_ values at temperature T in °C.
2. *Fit the free parameters such that the relative difference between measured (SS_exp_) and simulated (SS_sim_) steady state metabolite concentrations of Glc, D-Gat, KDG and Gly become minimal (Fig 2, Eq 2)*.
  $$\frac{SSsim-SSexp}{SSexp}$$ (2)

The pseudo-code of the optimization algorithm is detailed in Materials and Methods, Parameter estimation.

Indeed, we could fit the free parameters such that:

$${SS}_{sim}^{80{}^{\circ}C}\approx {SS}^{80{}^{\circ}C}$$ (3)

Eq 1 is approximattley fulfilled with a ratio between both steady states of:

$$\frac{{SS}^{80{}^{\circ}C}}{{SS}_{sim}^{80{}^{\circ}C}}=1.008$$ (4)

S2 Fig shows the simulated metabolite and flux steady state values for 70°C and 80°C.

### 3. Model validation

**Fig 3:**
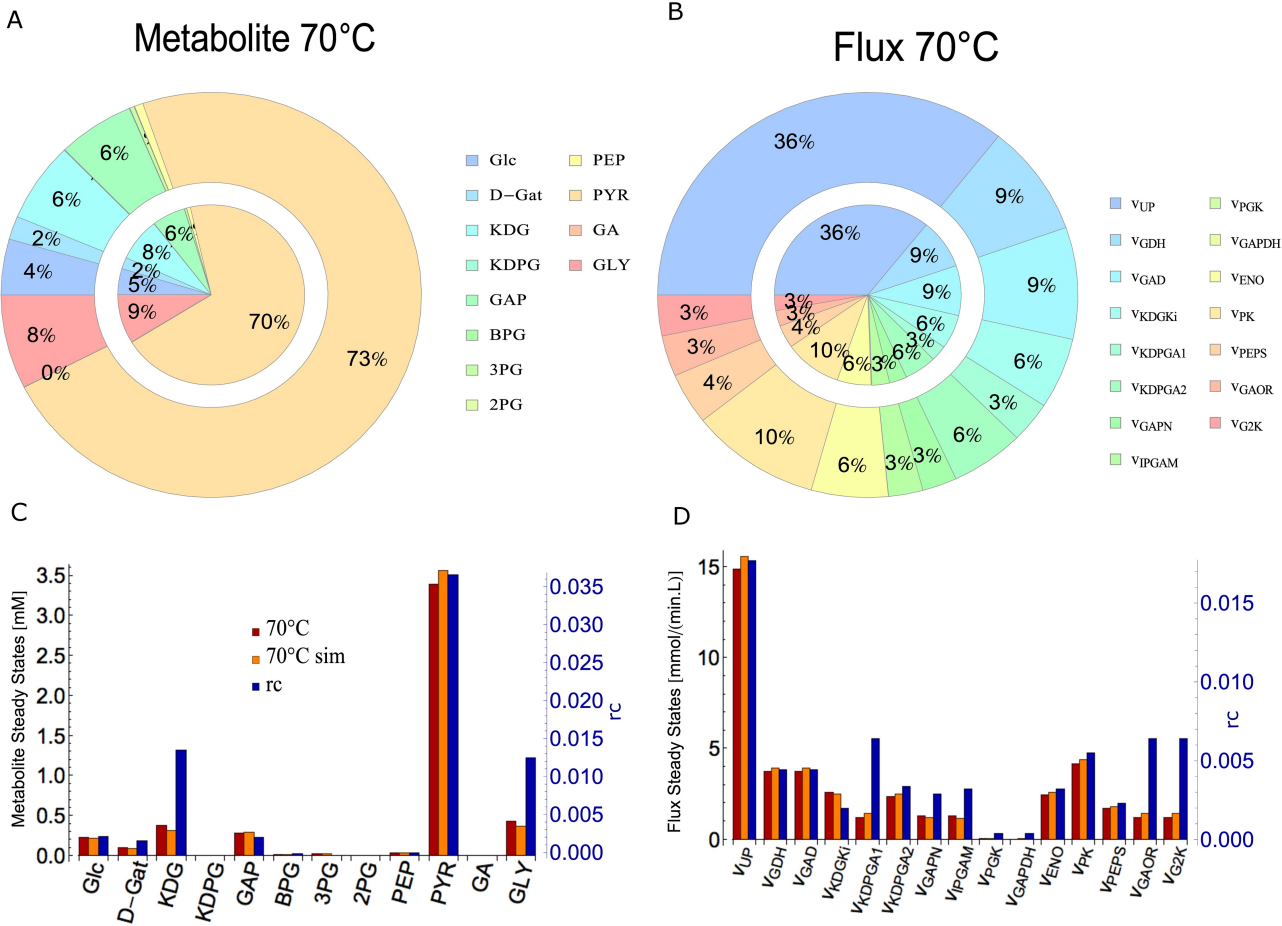
Model validation. Comparison between ${SS}_{sim}^{70{}^{\circ}C}$ and *SS*^*70°C*^ for metabolite and flux steady states. A-B) Pie Chart. Inner circle: percentual contribution of each metabolite (A) or flux (B) steady states to the overall steady state at 70°C (*SS*^*70°C*^); Outer circle: percentual contribution of each metabolite (A) or flux (B) steady states to the overall steady state at 70°C (${SS}_{sim}^{70{}^{\circ}C}$) (0% contributions ignored); C-D) Bar Chart comparing the metabolite and flux steady states for ${SS}_{sim}^{70{}^{\circ}C}$ (Orange Bars), *SS*^*70°C*^ (dark red bars) and the relative ratio between ${SS}_{sim}^{70{}^{\circ}C}$ and *SS*^*70°C*^ (blue bars). The left y-axis refers to the ${SS}_{sim}^{70{}^{\circ}C}$ and *SS*^*70°C*^ and the right y-axis (blue) refers to the relative ratio rc. C) Metabolite; D) Flux.

We validated the model by applying the same strategy as in the parameterization, but with different data sets: we halved the velocity parameters at 80°C and compared the obtained steady states (${SS}_{sim}^{70{}^{\circ}C}$) with the parameterized 70°C model (*SS*^*70°C*^). Fig 3 shows the metabolite and flux values between *SS*^*70°C*^ and ${SS}_{sim}^{70{}^{\circ}C}$, as well as their weighted relative changes *rc* (Eq 5).

$$rc=\frac{\left({SS}_{sim}^{70{}^{\circ}C}-{SS}^{70{}^{\circ}C}\right)}{{SS}_{sim}^{70{}^{\circ}C}}*\frac{E_{i}}{\sum E}$$ (5)

where *E*_*i*_/(∑*E*) is the relative weight of each metabolite or flux *E*_*i*_ in the overall network. *rc* values vary between -0.01 and 0.04 (metabolites, Fig 3A) and between -0.2 and 0.01 (fluxes, Fig 3B). Moreover, the average ratio between ${SS}_{sim}^{70{}^{\circ}C}$ and *SS*^*70°C*^ metabolites and fluxes is 1.01 and 1.29, respectively. The agreement between simulations and independent experiments is approximately 1:1. (S3 Fig shows the metabolite and flux values between ${SS}_{sim}^{80{}^{\circ}C}$ and *SS*^*80°C*^).

To further validate the model, we simulated a knockout of the spED branch and compared the estimated ratios for Glc, D-Gat, KDG and Gly with the experimentally measured metabolite ratios for the corresponding knockout strain PBL 2025ΔSSO3195 (spED = 0) and for the WT (Table 1). This simulation has a good agreement for Glc, D-Gat, GA and Gly and a lower agreement for KDG.

**Table 1:** Metabolome ratios for Glc, D-Gat, KDG, GA and Gly for the WT and knockout. Measured and simulated metabolite ratios of the ED pathway between PBL 2025ΔSSO3195 (spED = 0) and WT at 80°C.

Metabolite	Experimental Ratio	Simulated Ratio	Relative difference [%]
**Glc**	1.54	1	-35
**D-Gat**	1.09	1	-8
**KDG**	32.7	2.79	-91
**GA**	6	2.79	-53
**Gly**	2.91	1.44	-53

Thus, our parameterized model gives consistent results and a very similar overall picture for 70°C and 80°C for both the simulated (${SS}_{sim}^{70{}^{\circ}C}$, ${SS}_{sim}^{80{}^{\circ}C}$) and the parameterized (*SS*^*70°C*^, *SS*^*80°C*^) steady states using data at respective temperatures (Fig 3) for the wild type. Furthermore, it complies with Van’t Hoff’s rule and shows agreements between simulations and independent experiments.

### 4. The modified ED pathway is robust to deterministic and stochastic perturbations

Organisms evolved to be resistant towards environmental variations [32]. In fact, there are theories, such as the theory of highly optimized tolerance (HOT), which argue that biological systems with greater level of complexity are, on the one hand, optimized to respond to certain perturbations and, on the other hand, susceptible to un-antecipated perturbations, such as infections [33, 34]. These perturbations constitute an *addition* to the biological system. There are other types of perturbation, however, that mimic the *deletion* of specific components of a network. For example, the two-branched HOG signaling pathway in yeast shows more robust and faster responses than single-branched engineered yeast [35]. The ability to survive in such conditions is usually termed as robustness [32, 36, 37].

Although a definition of biological robustness in difficult to attain [38], Stelling *et al*. propose that robustness is “the ability to maintain performance in the face of perturbations and uncertainty” [39]. These perturbations can be internal (model parameters) or external (input conditions), or can be the lack of a module or component (deletion).

In the particular case of the ED pathway of *S*. *solfataricus*, the lack of a module corresponds to the knockout of a specific branch. We tested the robustness of this biological system when faced with internal and external perturbations. For this purpose, we used a deterministic and a stochastic approach.

#### Deterministic approach: Single parameter sensitivity analysis

To test the robustness of the system to single parameter changes, we calculated the sensitivity (S) of each metabolite to changes of 20% in one V_max_ parameter at a time. Calculation details in S1 Analysis. A sensitivity of S = 1 indicates direct proportionality of a change in a parameter and the considered output. Values of S below two can be considered unsensitive.

**Fig 4:**
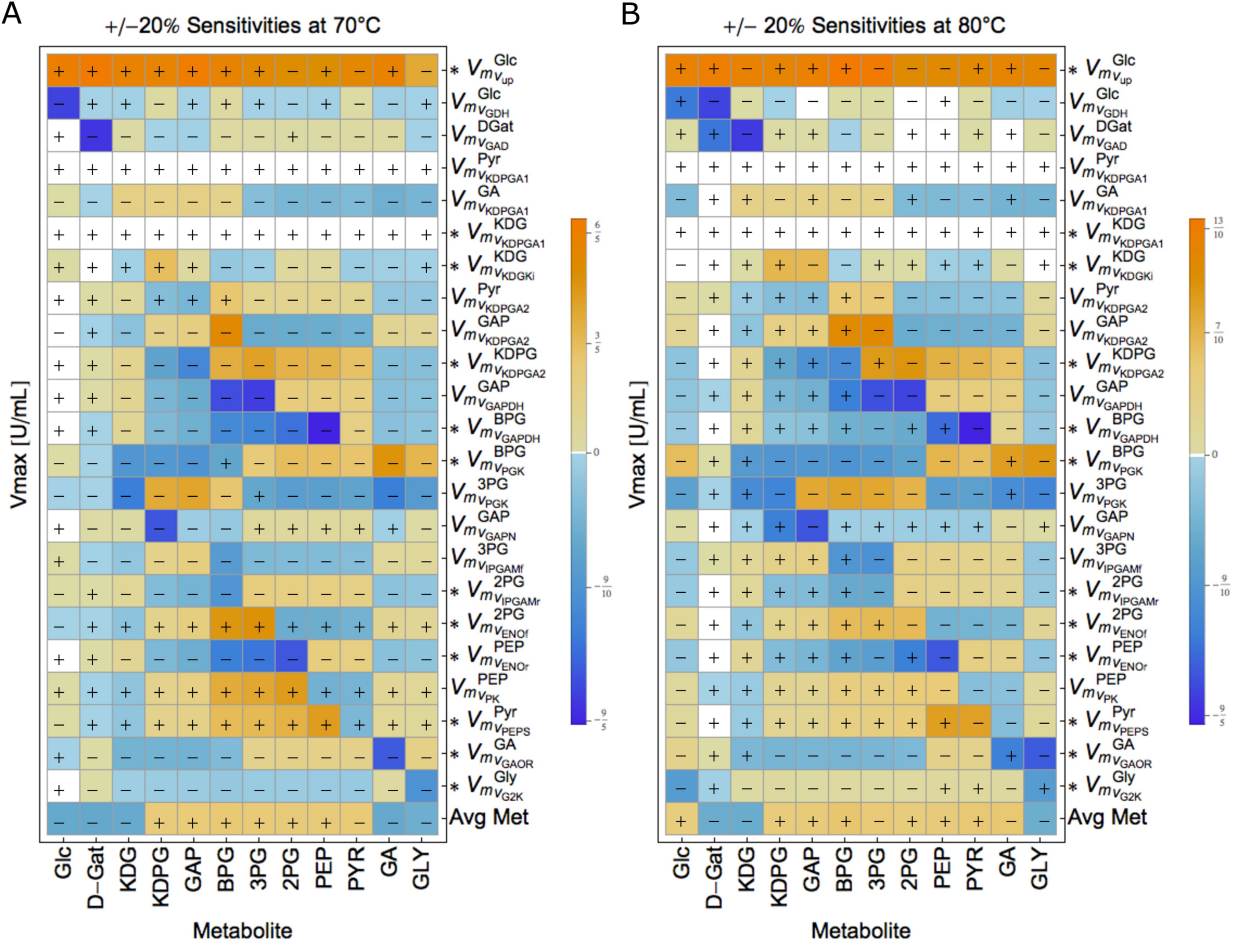
Sensitivity analysis. Matrix plot of the maximal sensitivities of the modified ED pathway in *S*. *solfataricus* at A) 70°C and B) 80°C. Columns represent the Metabolites and rows represent the V_max_. “+”and “-”indicate whether the maximal sensitivity was obtained for +20% or -20% variation, respectively.

We calculated the steady state metabolite concentrations with respect to perturbations in V_max_ values, which are the most likely to change with temperature (Fig 4).

The system has low sensitivivity to single parameter perturbations both at 70°C and 80°C. In fact, the maximal sensitivity is of S = -1.8 and reflects the influence of ${Vm}_{vGAPDH}^{BPG}$ on Pyr.

To further investigate the general sensitivity of a metabolite towards changes in V_max_, we computed the average sensitivity of each metabolite over all parameters (Fig 4, bottom). This average lays between -1<S<1 at 70°C and 80°C, which indicates that the system is overall robust to single (V_max_) parameter perturbations at 70°C and 80°C.

The overall input-output behavior of the system is insensitive to perturbations both at 70°C and 80°C.

#### Stochastic approach: Monte Carlo analysis

**Fig 5:**
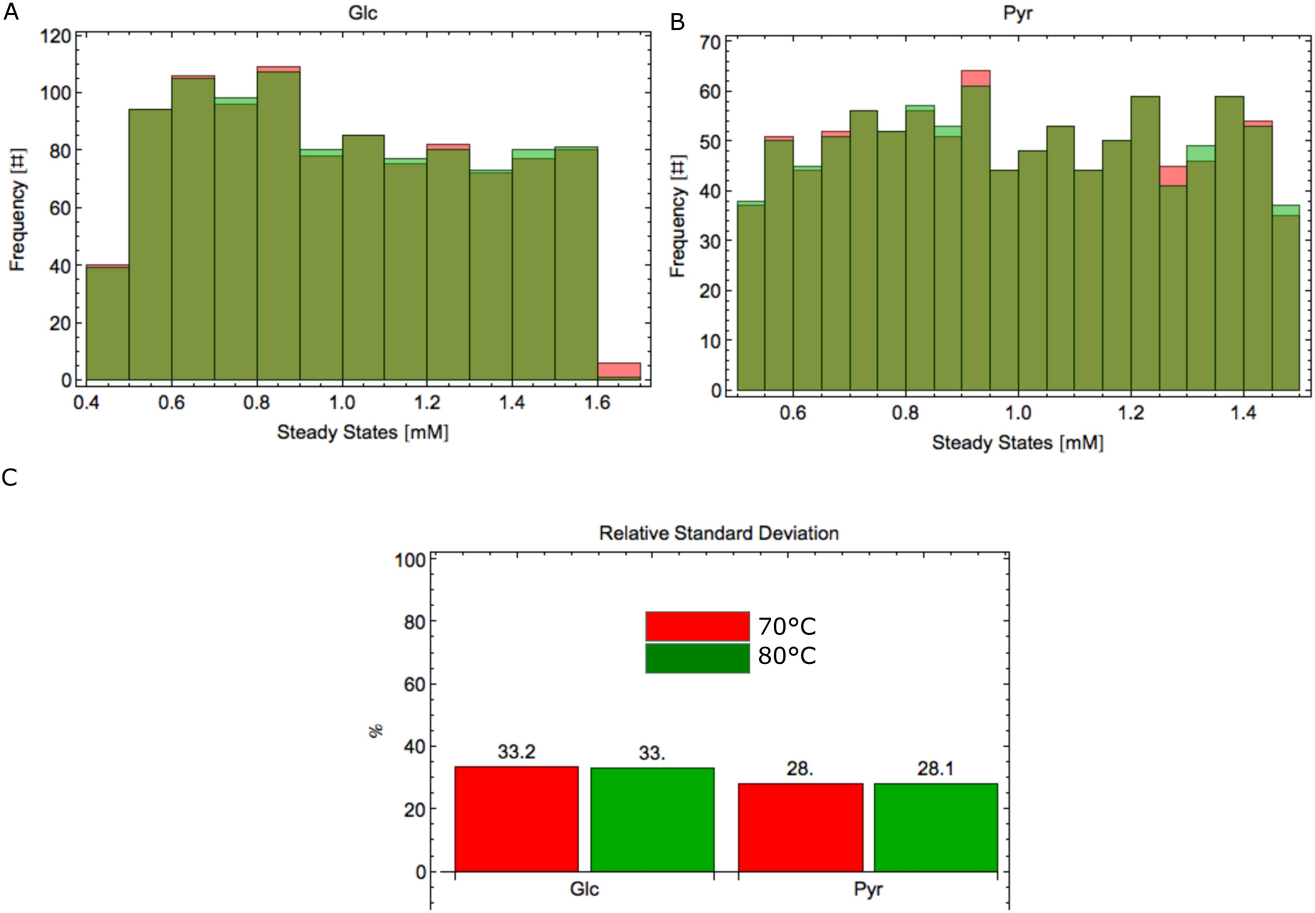
Stochastic external perturbations. Histograms of Glc, (A), and Pyr (B), and the relative standard deviations (C) of these three metabolites, at 70°C and 80°C, when ${Vm}_{vUp}^{Glc}$ changes 50% around its original value. Red bars represent the model at 70°C and green ones at 80°C.

**Stochastic external perturbations:** We perturbed the input of the system, ${Vm}_{vUp}^{Glc}$, 50% around its original value. We applied stochastic perturbations using the Monte Carlo method (further details in the Materials and Methods section). We computed how changes in ${Vm}_{vUp}^{Glc}$ will change the steady state of Glc (the first metabolite of the network), and Pyr (the output of the network). Fig 5 shows the histograms for Glc, (A), and Pyr (B), and the relative standard deviations (RSD) (S1 Text) of these three metabolites, at 70°C and 80°C.

Changes of 50% in ${Vm}_{vUp}^{Glc}$ imply a dispersion of 33% in Glc and 28% in Pyr at 80°C. This means that the system has a slightly tighter regulation mechanism in terms of the system output (Pyr) as compared with the input metabolite Glc.

**Stochastic internal perturbations:** We perturbed all reaction velocities (V_max_ and V_deg_) in the model and checked how these perturbations affected the steady state of each metabolite. Again, we performed this study for the model parameterized for 70°C and 80°C. We conducted a Monte Carlo Analysis by simultaneously sampling experimentally measured velocities ($V_{max}^{meas}$) within their measured standard deviation or around 20% of their original value using a uniform distribution for the fitted velocities ($V_{max}^{free}$).

We calculated new steady states using these stochastically generated sets of $Vmax=(V_{max}^{meas}+V_{max}^{free})$ parameters and analysed their respective RSDs.

**Fig 6:**
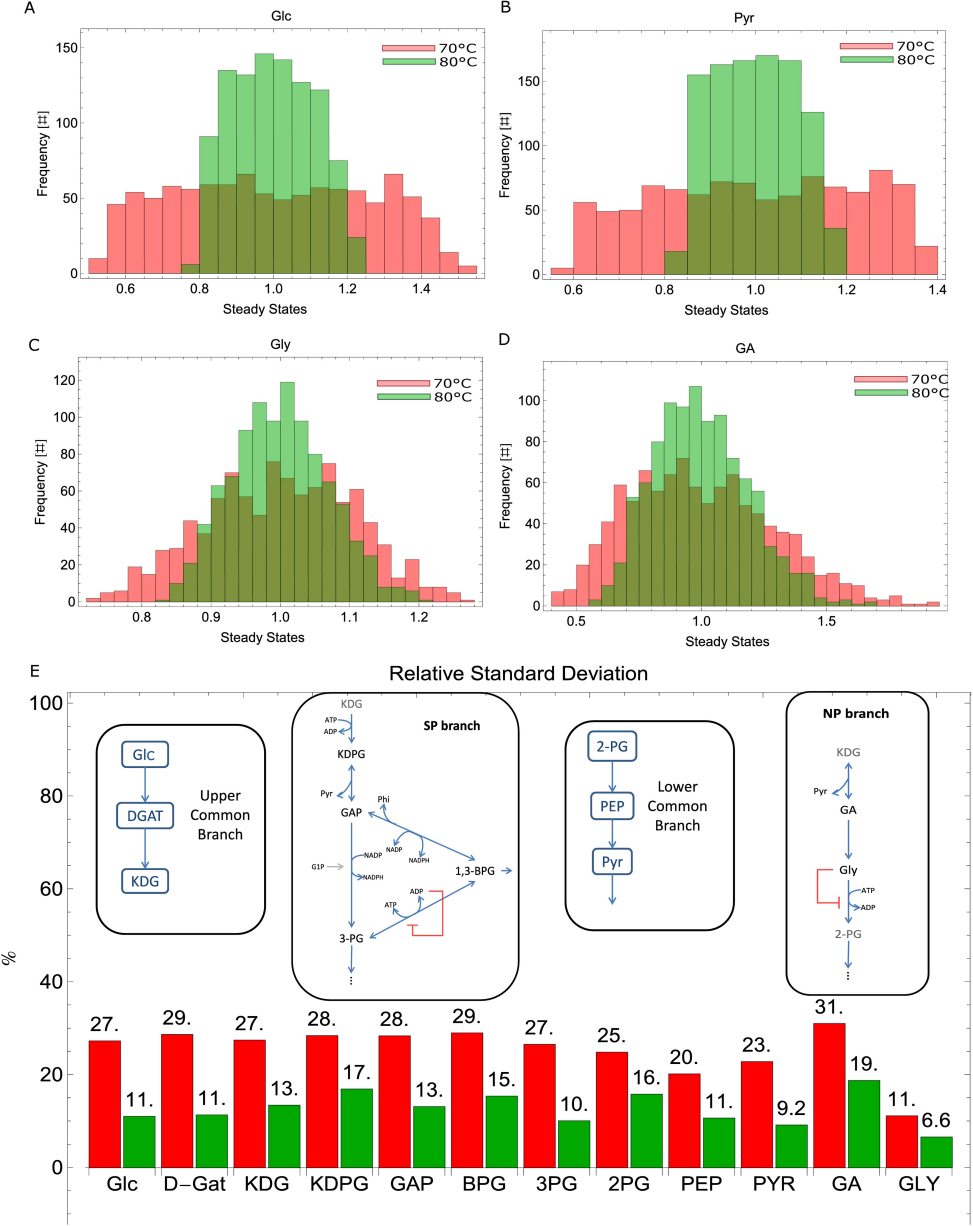
Stochastic internal perturbations. Histograms accounting for metabolites’ relative steady state variations after stochastically varying V_max_. Red: 70°C, Green: 80°C. A) Glc; B) Pyr; C) Gly; D) GA; E) RSD and network elements. # means counts.

Remarkably, the RSD of Pyr decreased by more than half, from 22% to 9%, when increasing the temperature from 70 to 80°C; nevertheless, Gly is the most robust metabolite to V_max_ perturbations, with 7% RSD at 80°C (Fig 6E). This metabolite from the npED branch controls its own catabolism by inhibiting the enzyme V_GK_, responsible for its degradation. On the other hand, GA (the metabolite upstream Gly), has the highest RSD (Fig 6E).

These results show that this mathematical model representing the ED pathway of *S*.*solfataricus* is more robust to stochastic internal perturbations at its ideal growth temperature, 80°C, than at 70°C.

#### The ED pathway robustly produces Pyr, when faced with deletion and uncertainty

The structure of the modified ED pathway, with two parallel branches that split at KDG and converge again at 2-PG, indicates modularity and redundancy in terms of system design, two characteristics of robust systems. In this case, redundancy refers to a component that is not strictly necessary to functioning, but can become necessary in case one other component fails. This system design method is widely used in engineering and has been observed in biological systems [35]. Moreover, whereas both branches are able to perform glycolysis, the spED branch seems to be preferred for gluconeogenesis since it would require additional energy investment at the level of the PGK reaction and proceeds via thermolabile intermediates. Therefore, only the spED branch seems to be indispensable.

To test if this metabolic network is robust to deletion when parameterized with experimental data of growth at 80°C, we simulated the knockout of either branch (SP = 0, NP = 0) and compared the model behavior with the wild type (WT), where the two branches are active:

1. *SP = 0: The V_max_ parameter of the reaction V_KDGKi_ is set to zero, to simulate the spED branch knockout*;
2. *NP = 0: The V_max_ parameters of the reversible reaction V_KDGPA1_ (acting on the npED branch) are set to zero, to simulate the npED branch knockout*;
3. *WT: The original model of the ED pathway (from Glc intake to Pyr production) that includes both arms of the pathway*.

KD(P)GA is a bifunctional enzyme active on KDPG (spED branch) and KDG (npED branch), meaning that, in experimental terms, the knockout of KD(P)G will automatically shunt not only the spED branch, but also the npED branch. Consequently, this experiment is not possible in the wet lab. However, using a systems biology approach, we can test *in silico* how the system behaves when the npED branch is inactive and the spED branch is active.

The ED pathway can produce Pyr when either the spED or the npED are set to zero. In fact, the npED branch reports to be mainly responsible for Pyr production, because when this branch is set to zero, the steady state levels of Pyr decreases (S4 Fig). Interestingly, when the spED branch is set to zero, the production of Pyr increases. Thus, a notable amount of carbon seems to be lost for Pyr production either through the degradation of the thermolabile metabolites (i.e. GAP, BPG) from the spED branch and/or the sink towards gluconeogenesis v_sinkGAP_. This indicates that 1) the spED branch can fine-tune the production of Pyr by potentially controlling the concentration of thermolabile intermediates and thus their decomposition and re-directing to gluconeogenesis (*v*_sinkGAP_) and 2) each branch alone can produce a substantial amount of Pyr.

**Fig 7:**
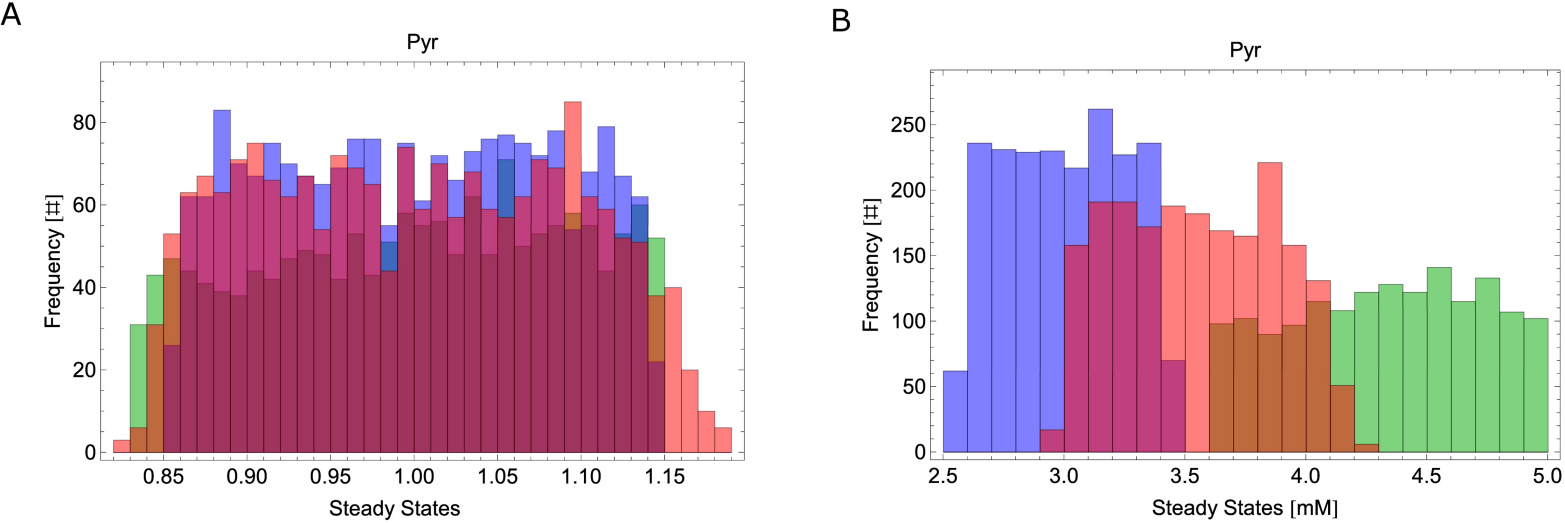
Robustness to uncertainty and branch deletion. Histogram accounting for uncertainty and branch deletion at the level of Pyr steady state. Blue bars represent the histogram of Pyr production when the npED branch is set to zero (NP = 0), green bars represent the histogram of Pyr production when the spED branch is set to zero (SP = 0), and red bars represent the histogram of Pyr production for the wild type (WT). A) Relative steady state values. B) Absolute steady state values. # represents counts.

To test the contribution of each branch to the overall robustness of the system, we computed the effect of Monte-Carlo perturbations at the level of Pyr steady state for the WT, spED = 0 and npED = 0. The results show that Pyr steady states (absolute value (Fig 7A) and RSD of Pyr steady states of all Monte Carlo iterations (Fig 7B)) are similarly robust in both single branch mutants and the wild type.

**Fig 8:**
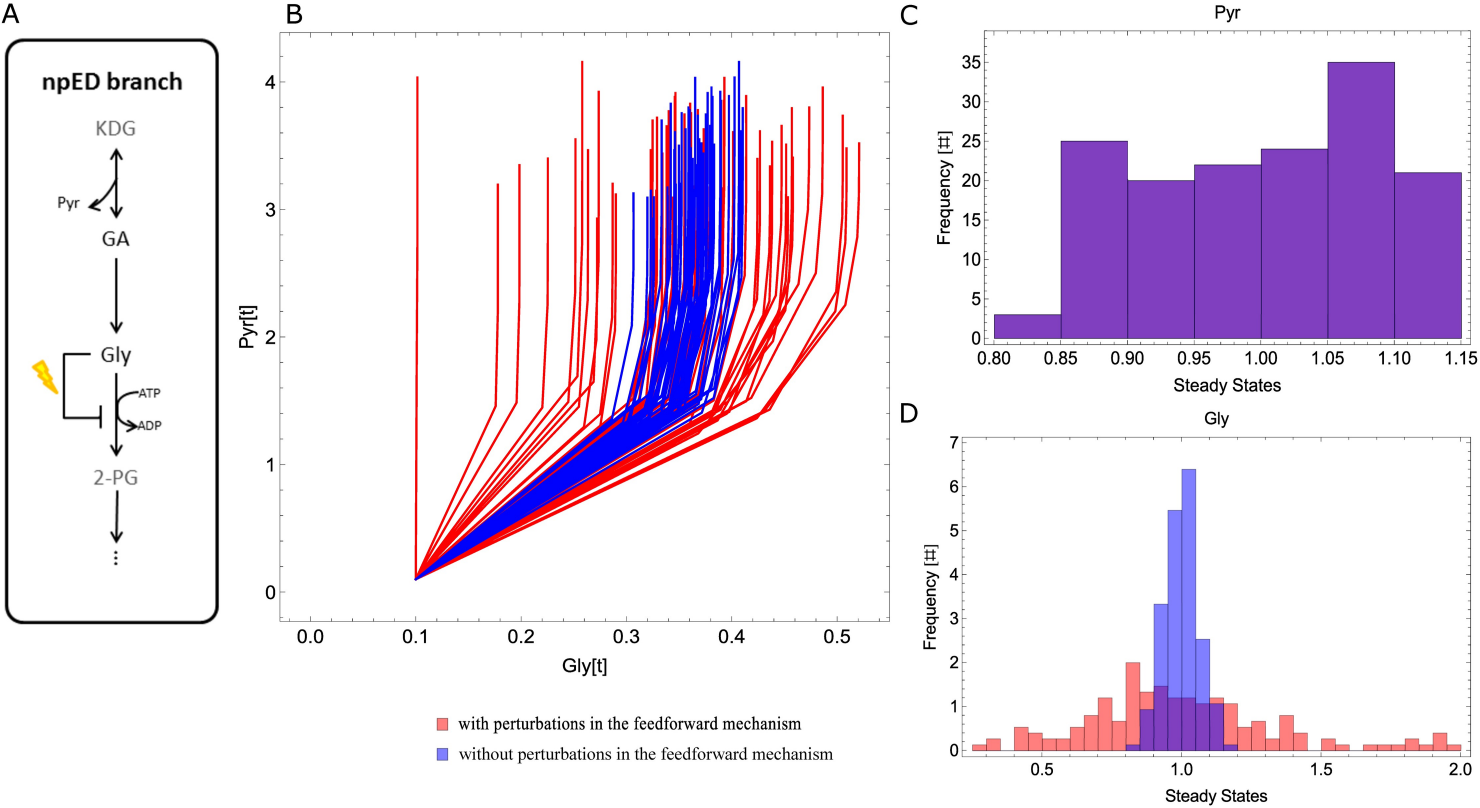
Perturbation of the negative feedforward reaction of the npED branch. Stochastic perturbations on the feedforward parameters affecting V_GK_. A) Pathway diagram of the npED branch with perturbation on the feedforward connection; B) Parametric plot relating Pyr and Gly with feedforward perturbations (red) and without feedforward perturbations (blue); C) Histogram of Pyr steady states with feedforward perturbations (red) and without feedforward perturbations (blue); D) Histogram of Gly steady states with feedforward perturbations (red) and without feedforward perturbations (blue).

To further investigate the systemic properties of Pyr production by the modified ED pathway in *S*.*solfataricus*, we investigated the role of the negative feedforward mechanism (of the npED branch) in terms of Pyr and Gly production in the WT model. The npED branch harbors a negative feedforward mechanism, where Gly controls its own conversion by inhibiting the activity of the enzyme V_GK_ [15]. To understand if this regulatory mechanism adds to the robustness of Pyr, we randomly perturbed the inhibition parameters of the reaction V_GK_ around 20% of their original value, in addition to the Monte Carlo perturbations imposed to V_max_. We checked the relationship between Gly and Pyr for two situations: 1) Monte Carlo perturbations of V_max_ (20%) and 2) Monte Carlo perturbations of V_max_ (20%) and the inhibition parameters of the reaction V_GK_ (20%). Fig 8 illustrates the analysis. We analysed further the feedforward mechanism acting on V_GK_ (S1 Analysis).

Perturbations on the feedforward parameters of v_GK_ alter the steady states of Gly (Fig 8B) and D) but not of Pyr (Fig 8C), indicating that this feedforward mechanism does not affect Pyr (S2 Analysis). The reaction V_GK_ functions as a throttle valve that controls the fluxes between the npED and the spED branch and guarantees that internal noise will not affect the production of Pyr. This has been shown experimentally by Kouril and colleagues [15] and our mathematical model supports this notion (Fig 8C).

Taken together, these results suggest that the modified ED pathway can deliver Pyr robustly when faced with deletion (spED or npED branch deletion) and uncertainty (random perturbations). Both branches alone can robustly deliver Pyr, however, carbon loss through the spED branch is diminished by re-directing part of the carbon through the npED branch, thereby increasing the overall Pyr production. Moreover, Gly works as a stabilizer that guarantees a robust delivery of Pyr in spite of perturbations.

## Discussion

*S*. *solfataricus* thrives in a hostile niche where few organisms can survive. The evolutionary strategies employed by this extremophile to survive in a hot and acid environment are not yet completely elucidated. Nevertheless, *S*. *solfataricus* is used as a model organism for resisting and surviving in extreme conditions, such as in simulated space environment [40]. In fact this study shows that short-term storage of *S*. *solfataricus* at temperatures from -196°C to 85°C do not change significantly the growth profile of this extremophile. Unlike the majority of organisms, glucose catabolism happens via the modified ED pathway, which has two parallel branches and involves novel biocatalysts. The role of these two branches has been investigated by Kouril and colleagues who suggest that the spED branch has a gluconeogenic function and the npED branch compensates for the deletion of the former, whereas the latter works as a control flux point via v_GK_ [15]. Other studies suggest that these two branches bring “metabolic thermoadaptation” to (hyper)thermophilic archaea [12, 16], because catabolism via the spED branch proceeds via thermolabile intermediates (i.e. GAP), which might result in carbon loss at high temperature [20].

Resistance to high temperatures and glucose metabolism via a branched pathway distinguish *S*.*solfataricus* from organisms of the other domains of life. To understand the role of these two assets within the evolutionary survival strategy of *S*.*solfataricus*, we investigated the ED pathway in terms of its robustness and ability to adapt to deletion and uncertainty, using a systems biology approach.

Systems biology aims at understanding the emergent properties of a biological mechanism by combining mathematical modelling with experiments [41]. This approach allows gaining new insights into the dynamic properties of the biological system that would not be revealed using solely an experimental approach.

Here, we present the first kinetically parameterized mathematical model of the *S*.*solfataricus* ED pathway, from Glc consumption to Pyr production. This model includes not only experimental data on enzyme activity, but has also been parameterized using metabolomics data. The parameterized model can correctly predict its behavior at 70°C, provided that Van’t Hoff’s rule holds. It can also predict the behavior of internal Glc and of the metabolites D-Gat, GA and Gly when faced with a knockout of the spED branch. This mathematical model can not only predict the behavior of single elements, but also systemic properties of the ED pathway. In fact, as shown in [15], the model supports that the reaction V_GK_ works as a stabilizer that controls the production of Pyr.

Several other studies have combined experimental data on enzyme kinetics with mathematical modelling to understand glycolysis in other organisms. Penkler and colleagues integrate the enzyme kinetics of the Embden–Meyerhof–Parnas pathway of *Plasmodium falciparum* in a mathematical model [42] and provide access to the model and data through the SEEK platform [43]. Yeast glycolysis has been tested in terms of its enzyme kinetics and also brought together in a mathematical model [44, 45]. In red blood cells, Mulquiney and colleagues present a detailed kinetic mathematical model, focusing on the regulation of 2,3-bisphosphoglycerate (2,3-BPG) metabolism [46]. When experimental procedures are not able to deliver the kinetic parameter values, a desired solution is to estimate those parameters in such a way that the biological properties of the overall network are kept consistent. However, the estimation of kinetic parameters is an arduous task and some authors have proposed methods to estimate non-measured kinetic parameters. Saa and Nielsen [47] present a framework that conveys the systematic parameterization and sampling of enzyme reactions and Liebermeister and Klipp [48] quantify uncertain parameters using probability distributions. Here, we use a heuristic approach, guided by the aims to achieve i) steady states; ii) consistent predictions upon temperature shifts based on Van’t Hoff’s rule and Arrhenius equations (Materials and Methods, Eqs (8–11); and iii) consistent predictions of metabolomics data. We present here a mathematical model of the branched *S*.*solfataricus* ED pathway, parameterized with *in vivo* enzyme kinetic data for different temperatures, which is able to consistently predict the metabolite steady states of the ED pathway upon temperature shifts. We used the validated model to study the behavior of *S*.*solfataricus* with respect to deletion and uncertainty. For that, we conducted a robustness (and sensitivity) analysis in different perspectives.

There are several mathematical models accounting for specific phenomena of metabolism in *S*.*solfataricus*. Kouril and colleagues determined *in vitro* specific reactions of gluconeogenesis of *S*.*solfataricus* and, with this data, reconstructed mathematically that pathway to show that intermediate instability at high temperature can perturb pathway performance [20]. In one other study, the role of the two ED branches is tested [15].

Our mathematical model is the first model that combines the complete carbon metabolism from Glc to Pyr and incorporates cell-free extract, half-life and metabolomics experimental data for most of the reactions.

First, we tested how *S*.*solfataricus* adapts to growth at different temperatures. This archaeon grows optimally at 80°C, nevertheless, experimental data show that it is able to grow at other temperatures. However, growth at 70°C is slower than growth at 80°C and the averaged enzyme kinetics follow the Van’t Hoff rule [49]. We conducted a sensitivity analysis, by varying each *V*_*max*_ parameter at once around 20% of the original Vmax value, and we checked its influence on each metabolite at 70°C and 80°C. Results show that the respective metabolites are insensitve to internal single parameter changes at both temperatures. *S*.*solfataricus* shows no sensitivity in the output (Pyr) when the input reaction (${Vm}_{vUp}^{Glc}:\;\to Glc$) changes. Following, we conducted a robustness analysis by 1) stochastically varying the Glc uptake rate (extrinsic perturbations) and 2) stochastically varying simultaneously all the velocity parameters of the network (intrinsic perturbations). When faced with extrinsic perturbations, the system is marginally more robust in terms of Pyr production, but there is no siginificant difference between 70°C and 80°C. When faced with intrinsic perturbations, the system is consistently more robust at 80°C not only in terms of Pyr production, but also in terms of all the intermediates of the ED pathway represented in this mathematical model. Together, this sensitivity and robustness analysis implies that *S*.*solfataricus* can robustly cope with single parameter perturbation and adapts more effectively to systemic, intrinsic stochastic changes when it grows at 80°C as compared with growth at 70°C, which can add to an explanation why it can out-compete other organismns in this environment.

Second, we used the mathematical model to analyze the role of the two branches of the ED pathway when parameterized with data at 80°C. For this purpose, we set the velocities of the first reaction of either branch to zero, and then checked the influence of each in the steady states of metabolites and fluxes. The spED branch knockout implies an ascent production of KDG, Pyr and Gly along with the activity of several fluxes of the npED branch. The npED branch knockout will increase KDG and GAP and decrease Pyr and Gly production. Then, to test the robustness of the system to deletion, we computed a Monte Carlo analysis to check how stochastic variations influence the steady states of Pyr when one module of the network fails. Knocking out the spED or the npED branch has a minimal effect on the robustness of the system, suggesting that the system is extremely robust, even under extreme conditions, such as its optimal growth temperature of 80°C. Indeed, the perturbation of the feedforward parameters present in the npED branch dramatically affects Gly production but does not affect at all Pyr delivery. With this study, we conclude that the ED pathway is extremely robust at 80°C, either when faced with deletion or with uncertainty and that v_GK_ works as a flux controller, making sure that the amount of Pyr remains constant despite perturbations (experimental result verifies the model) [15].

In evolutionary terms, the cost of maintaining two parallel branches comes with the benefit of robustness to deletion and uncertainty. More specifically, why maintain the dispensable npED branch? Our mathematical model shows that, in fact, *S*.*solfataricus* has maintained the two branches of the ED pathway as a survival strategy to endure in a hostile environment, supporting a model in which both branches substantially contribute to pyruvate production under sufficient internal glucose levels. However, pyruvate production through the essential spED branch is subject to carbon loss by thermolabile intermediates and a certain flux to gluconeogenesis. The model supports a role of the npED branch to partly diminish this carbon loss by re-directing some of the carbon to the npED branch, where no such losses are expected. This increase might be sufficient to grant an evolutionary advantage and to justify the additional costs. This strategy encompasses robustness to internal perturbations along with the lack of one branch and adaptation to habitat changes, embodied by the ability to grow when faced with temperature changes.

## Materials and methods

Mathematical models were implemented in Wolfram Mathematica version 9. ODEs were solved using the command NDSolve. The steady states were calculated by running the simulations over a long period of time and testing if the rate of variation of the ODE variables (metabolites) did not change more than 10^−6^. This method can be applied to this particular system, because it does not present oscillations.

Extrinsic stochastic perturbations: Monte Carlo analysis was done by stochastically varying the input (Glc uptake rate) 50% around their original value and sampling them 1000 times from a Uniform Distribution.

Intrinsic stochastic perturbations: Monte Carlo analysis was done by stochastically varying all the V_max_ parameters of the network around their standard deviation (for experimentally measured V_max_) or 20% around their original value (for predicted V_max_) and sampling them 1000 times from a Uniform Distribution.

### Available data

#### Enzyme activities

V_max_ measurements using cell-free (or crude) extracts from *S*. *solfataricus* give a better picture of *in vivo* enzyme activities than *in vitro* measurements using artificial enzyme amounts. To scale the cell-free extract enzyme activity to a single cell, we assumed a protein concentration of 250 g/L cytosol to convert U/mg into mM/min [44]. However, enzyme concentrations are likely to change upon temperature shifts and, therefore, specific activities of enzymes of the ED were determined at 70°C and 80°C (assay temperature) in cell-free extracts of *S*. *solfataricus* grown cells at 70 and 80°C, respectively. We incorporated the scaled enzyme activities in the mathematical model as V_max_ values in the respective fluxes (see Materials and Methods section). Notably, the measured enzyme activities comply with the Van’t Hoff rule on average, i.e. they double with a temperature increase of 10°C (S2 Fig).

Available *V*_*max*_ parameters were set to the corresponding measured or published value and multiplied by an internal volume factor per mg protein of 250 g/L cytosolic volume. Enzyme activities (EA) were measured as U/g, where U = μmol/min. Fluxes in cells were considered in mM/min. To scale the enzyme activity to a single cell, a factor of cytosolic volume of 0.250 g/mL was incorporated in the EA parameters, so that EA*volFactor = [μmol/min/g*g/mL] = >[μmol/(min mL)].

#### K_m_ and K_i_ constants

K_m_ and K_i_ determination in cell-free (crude) extracts was not possible due to the low enzyme activities. Since there were no temperature dependent values available for Michaelis-Menten constants (K_m_) and inhibitory constants (K_i_), we used previously published data. Furthermore we assume—in this particular mathematical model–for simplification and due the lack of more specific data, that K_m_ and K_i_ are temperature-independent. We use, for the calculations in this mathematical model, the published K_m_ and K_i_ values even though, in some of the cases, these values were measured at different temperatures.

#### Half lifes

We included available half-life values for the metabolites PEP, GAP and 1,3-BPG [2, 20, 31] as degradation rates ($V_{half}^{meas}$). The half-life of 1,3-BPG at 80°C was estimated from the available measured data (Eq 6).

$$V_{deg}^{BPG}(80{}^{\circ}C)=V_{deg}^{BPG}(70{}^{\circ}C)/Mean\frac{V_{half}^{meas}(70{}^{\circ}C)}{V_{half}^{meas}(80{}^{\circ}C)}$$ (6)

Half lifes were converted to degradation rates [1/min]. K_m_ in [mM].

#### Metabolomics data

We parameterized the model with measured metabolite ratios of Glc, D-Gat, KDG and Gly between 70°C and 80°C [50].

These metabolomics data were used for Glc, D-GAT, KDG and Gly—determined for the WT and spED branch knockout at 70°C and 80°C, respectively.

### Parameter estimation

The model has the following distinct sets of temperature-dependent parameters: measured V_max_ values $V_{max}^{meas}$, measured half-lives $V_{half}^{meas}$, free V_max_ values $V_{max}^{free}$ and free sink reactions $V_{sink}^{free}$.

We fitted the free parameters such that Eq 3 is verified and that measured metabolite ratios of Glc, D-Gat, KDG and Gly at 70°C and 80°C [50] are recapitulated (Fig 2 and Results section). To this end, we used the following optimization algorithm described in pseudo-code:

Minimize the sum of the respective squared residuals(SSR)(Eq 7):

$$SSR\left(V_{max,80}^{free},V_{sink,80}^{free},V_{max,70}^{free},V_{sink,70}^{free}\right)={\left(\frac{SS^{80{}^{\circ}C}\left(V_{max,80}^{meas},V_{max,80}^{free},V_{half,80}^{meas},V_{sink,80}^{free}\right)-SS_{sim}^{80{}^{\circ}C}\left(2V_{max,70}^{meas},2V_{max,70}^{free},V_{half,80}^{meas},V_{sink,80}^{free}\right)}{SS^{80{}^{\circ}C}}\right)}^{2}+{\left(\frac{\frac{Glc_{70}}{Glc_{80}}-0.97}{0.97}\right)}^{2}+{\left(\frac{\frac{DGat_{70}}{DGat_{80}}-1.27}{1.27}\right)}^{2}+{\left(\frac{\frac{KDG_{70}}{KDG_{80}}-4.16}{4.16}\right)}^{2}+{\left(\frac{\frac{Gly_{70}}{Gly_{80}}-1.91}{1.91}\right)}^{2}$$ (7)

Where numbers indicate measured ratios.

Such that:

1. *SS*^*70°C*^, ${SS}_{sim}^{70{}^{\circ}C}$, *SS*^*80°C*^, ${SS}_{sim}^{80{}^{\circ}C}$ are in steady state
2. $V_{max}^{meas}-SD{<V}_{max}^{meas}<V_{max}^{meas}+SD$
3. ${10<V}_{max}^{free}<1000\;\left[\frac{U}{g}CE\right]$
4. *SS*^*Metabolite*^ < 100 *mM*

Glc_70/80_, DGat_70/80_, KDG_70/80_ and Gly_70/80_ indicate simulated steady state metabolite concentration of Glc, D-Gat, KDG and Gly at 70°C and 80°C, respectively.

To have additional flexibility in the parameter estimation, we allowed the measured v_max_ values to vary within their measured standard deviation. The free v_max_ values were constrained to vary between 10 and 1000 U/g CE and simulated steady states were constrained to be lower than 100 mM.

This optimization algorithm was implemented with the function NMinimize of Wolfram Mathematica 9.

Metabolite initial conditions were all set to 0.1 mM, except for Glc and D-Gat which were set to the calculated steady state concentration derived from the kinetic rate laws (S2 Dataset). Variation of initial conditions did not change the final metabolite steady states.

We performed an identifiability analysis to the input parameters of the model, ${Vm}_{vUp}^{Glc}$, at 70°C and 80°C. Results show that ${Vm}_{vUp}^{Glc}$ is identifiable both at 70°C and 80°C S3 Analysis).

### Influence of temperature

We used the Arrhenius equation to incorporate the influence of temperature in the mathematical model. The Arrhenius equation is described by Eq 8:

$$k_{i}=A_{i}e^{-\frac{E_{i}}{RT}}$$ (8)

Where *k*_*i*_ represents the rate constants of the enzyme catalyzed processes including k_cat_. *E*_*i*_ is the activation enthalpy; *R* is the gas constant and *T* is the temperature in Kelvin. *A*_*i*_ is the pre-exponential factor. We want to analyze the effect of temperature in the system when it changes from *T*_*1*_ = 70 to *T*_*2*_ = 80 [°C] (*T*_*1*_ = 343.15 and *T*_*2*_ = 353.15 [K]), i.e., *T*_*2*_ = *T*_*1*_+10. Eqs 9–11 describe this process.

$$k_{i70}=A_{i}e^{-\frac{E_{i}}{R.T_{1}}}$$ (9)

$$k_{i80}=A_{i}e^{-\frac{E_{i}}{R(T_{1}+10)}}$$ (10)

The ratio between both yields:

$$R_{7080}=\frac{k_{i70}}{k_{i80}}=\frac{e^{-\frac{E_{i}}{R.T_{1}}}}{e^{-\frac{E_{i}}{R(T_{1}+10)}}}=1.817$$ (11)

*E*_*i*_ may be considered as temperature-independent constant and with characteristic activation energy of 60 kJ/mol. This yields *R*_*7080*_ = 1.817, and is in accordance with the Van’t Hoff rule (the velocity of a reaction doubles when the temperature increases in 10°C). Our experimental data on enzyme activity further supports this. In fact, the average of the enzyme activities at 80°C is the double of the average of the enzyme activities at 70°C (S2 Dataset). Therefore, we considered that the enzyme activity used in this mathematical model accounts for the temperature compensation based on the Arrhenius equation.

### Model validation

After the incorporation of the experimental data at 70°C and 80°C and achieving a steady state, we set the V_max_ values at 80°C to its half to simulate the behavior of the system at 70°C, using the Van’t Hoff rule. We named this model occurrence as “70°C sim”. Then, we compare the steady states of metabolites and fluxes at 70°C with “70°C sim”. We then computed the steady states of Glc, Gly, GA and KDG when the spED branch is knocked out and compared the model results with independent metabolome data measured for the strain PBL 2025ΔSSO3195 (spED branch knockout strain, [50], Table 1).

### Experimental data

#### *In vitro* assays with *S*. *Solfataricus* cell-free extracts

To study the effect of different growth-temperatures on the enzyme level, cell-free extracts were prepared from *S*. *solfataricus* cells grown on glucose under standardized growth conditions at 70 or 80°C, respectively, as described previously [49, 51]. *S*. *solfataricus’* original strain were ordered form the DSMZ in Braunschweig (DSM 1617, https://www.dsmz.de/catalogues/details/culture/DSM-1617.htm). Briefly, cells were resuspended in 0.1M HEPES/KOH buffer pH 7 at RT (0.5 g cells/ 1.5 ml buffer) containing 5mM DTT and 1-times complete protease inhibitor (Roche). Cells were disrupted by 4-times sonification (2 min, interrupted by 1 min cooling on ice, 5 min, amplitude 50%, time cycle 0.5). Cell debris was removed by centrifugation (16.000 x g, 45 min, 4°C) and the resulting supernatant dialyzed (Spectra/Por^®^ Dialyse Membrane MWCO: 3,5 kDa (Spectrum Laboratories, Inc.)) over night at 4°C against 0.1 M HEPES/KOH buffer (pH 7, RT) containing 5 mM DTT. The protein content was determined using the Bradford assay [52]. Subsequently, the obtained cell-free extract was used for the respective enzyme assay. For each temperature 3-independent *S*. *solfataricus* cultures were analysed and for the different enzyme assays three independent measurements as well as controls (e.g. by omitting either substrate or cell-free extract) were performed. Cells grown at 70°C and 80°C were assayed at their respective growth temperature, i.e. 70°C and 80°C respectively.

#### Enzyme assays using cell-free extracts (CE) of *S*. *solfataricus*

All except three enzyme activities of the ED pathway could be determined in cell-free extracts of *S*. *solfataricus* grown at 80°C (S2 Dataset). The enzymatic activities were measured in triplicate at 70 and 80°C by using 3 independent biological replicates (*S*. *solfataricus* cultivations) and specific activities (V_max_ values) were determined (S2 Dataset). The activities of KDG kinase (KDGK), glycerate kinase (GK), enolase (ENO) could not be detected in the cell-free extracts, probably due to an activity below the detection limit. However, characterization of the recombinant *S*. *solfataricus* enzymes produced in *E*. *coli* was reported previously [15, 53].

#### Glucose dehydrogenase (GDH)

GDH activity was measured in a continuous enzyme assay containing 0.1 M HEPES/KOH (pH 6.5 at at the respective assay temperature), 10 mM MgCl_2_, 10 mM NADP^+^, and 300 μg CE in a total volume of 500 μl. Reactions were started by the addition of glucose (final concentration 10 mM). Enzymatic activity was followed by monitoring the increase of NADPH at 340 nm.

#### Gluconate dehydratase (GAD)

GAD assays were performed as previously described in [49].

#### Bifunctional 2*-*keto*-*3*-*deoxy*-(*6*-*phospho)gluconate/glactonate aldolase (KD(P)GA)

KD(P)GA activity was determined in a discontinuous enzyme assay in 400 μL total volume, performed in 0.1 M HEPES/KOH (pH 6.5, at the respective assay temperature) containing either 30 mM glyceraldehyde (GA), 60 mM pyruvate or 40 mM GAP, 40 mM pyruvate and 350 μg CE. Reactions were started by the addition of one substrate (GAP or GA). The assay mix was incubated in a thermoblock at 70 or 80°C, and after 0, 2.5, 5, 7.5 and 10 min of incubation, 25 μL aliquots were withdrawn, respectively. The reaction was stopped by cooling the samples on ice and by adding 2.5 μL of 12% (w/v) trichloroacetic acid. Enzymatic activity was measured by using a modified TBA assay as described above.

#### GAP dehydrogenase (GAPDH)

GAPDH activity was measured in a continuous enzyme assay, monitoring the increase of NADPH at 340 nm. The assay was performed in 0.1 M HEPES/KOH (pH 6.5 at at the respective assay temperature), containing 1mM NADP^+^, 150 μg CE and 300 mM sodium arsenate in a total volume of 500 μl. Reactions were started by the addition of the substrate (10 mM GAP).

#### Non-phosphorylating GAPDH (GAPN)

GAPN assays were performed as previously described in [49].

#### Phosphoglycerate kinase (PGK)

PGK activity was measured in a continuous enzyme assay monitoring the decrease of NADH at 340 nm. The assay was performed in 0.1 M Tris/HCl (pH 6.5, at the respective assay temperature) containing 0.2 mM NADPH, 20 mM ATP, 25 μg recombinant *S*. *solfataricus* GAPDH (20 μl of a 80°C heat precipitated fraction), 0.02 M MgCl_2_ and 375 μg of CE in a total volume of 500 μl. Reactions were started by the addition of 3PG (final concentration 15 mM).

#### Phosphoglycerate mutase (IPGAM)

PGM activity was measured in a discontinuous enzyme assay at 80°C. The assay was performed in 0.1 M HEPES/KOH (pH 6.5 at the respective assay temperature) containing 0.1 M MgCl_2_ and 1 mg CE in a total volume of 500 μl. Reactions were started by the addition of 3PG (final concentration 20 mM). Detection of the produced 2PG was performed in a second step at 37°C in 0.1 M HEPES/KOH (pH 7, RT) containing 0.5 mM NADH, 5 mM ADP, 10 mM MgCl_2_, 4 U PK, 4 U LDH, and 4 U ENO (rabbit muscle, EC 4.2.1.11, Sigma-Aldrich). Enzymatic activity was followed by monitoring the decrease of NADH at 340 nm.

#### Enolase (ENO)

Enolase (SSO0913) catalyses the interconversion of 2-PG to PEP. Enzymatic activity was measured in catabolic direction (conversion of 2-PG to PEP) using pyruvate kinase and L-lactic dehydrogenase (LDH) as auxiliary enzymes. The continuous enzyme assay was performed at 50°C (total volume of 500 μl) in 100 mM HEPES/KOH, pH 6.5 (50°C), 10 mM MgCl_2_, 2 mM ADP, 0.5 mM NADH, 10 U PK, 6.5 U LDH, 0.16 mM or 0.28 mM 2-PG, respectively and 3.68 μg of enolase. Enzymatic activity was followed by monitoring the decrease of NADH at 340 nm.

For characterisation purposes, PEP and 2-PG formation due to enolase activity was followed photometrically in a continuous assay from 50°C to 88°C at a wavelength of 240 nm according to Warburg and Christian [54]. The assay was performed in 0.1 M HEPES/KOH (pH 6.5 at 80°C),10 mM MgCl_2_, 2 μg of enolase in a total volume of 500 μl. Reactions were started by the addition of 2PG/PEP (0,01–5 mM). The produced PEP is detected directly at 240 nm in 0.1 M HEPES/KOH (pH 6.5 at the respective temperature).

#### Pyruvate kinase (PK)

PK activity was measured in a discontinuous enzyme assay in 0.1 M HEPES/KOH (pH 6.5 at 80°C) containing 300 μg CE in a total volume of 120 μl. Reactions were started by the addition of PEP (final concentration 25 mM). Detection of the produced pyruvate was performed in a second step at 37°C and 340 nm in 0.1 M HEPES/KOH (pH 7, RT) containing 0.5 mM NADH, 5 mM ADP, 10 mM MgCl_2_, 4 U LDH.

#### 2*-*keto*-*3*-*deoxy*-*gluconate/galactonate kinase (KDGK)

KDGK activity was assayed in a continuous assay (total volume 500 μL) in the presence of 2 μg cell-free extract, 10 and 20 mM KDG/Gal, 5 mM ATP, 10 mM MgCl_2_, 5 mM NADP^+^, 0.1 M HEPES/KOH (pH 6.5, 80°C) and 2 μg *Sso*-GAPN (after heat-precipitation, see 2.6.3) and 50 μg of *Ttx*-KD(P)GA (fraction after heat-precipitation) as auxillary enzyme, monitoring the formation of KDPG. However, it has to be considered that the *Ttx*-KD(P)GA is also able to directly metabolize the substrat of this enzyme assay (KDG/Gal) to GA and pyruvate.

#### Glycerate kinase (GK)

GK activity in cell-free extracts was determined in a discontinuous test at 80°C, which monitors the glycerate-dependent formation of ADP. The phosphorylation of glycerate by ATP was followed by coupling the formation of 2PG to the oxidation of NADH via enolase (rabbit muscle), PK (rabbit muscle) and LDH (rabbit muscle) at 37°C. The GK assay was performed in 0.1 M HEPES/KOH (pH 6.5, 80°C) in the presence of 1 mg crude extract, 10 and 20 mM ATP, 5 mM EGTA and 20 mM MgCl_2_. The reaction was started by the addition of 5 mM D-glycerate and stopped after 0, 1, 2, 3, 4 and 5 min of incubation time on ice. The indicator reaction (500 μL total volume) was performed in 0.1 mM HEPES (pH 7, RT), containing 10 mM MgCl_2_, 5 mM ADP, 0.5 mM NADH, 3.5 units of PK (rabbit muscle), 2.5 units of LDH (rabbit muscle) and 100 μL aliquots from the GK assay. The reaction was started by addition of 1 U enolase (rabbit muscle).

#### Phosphoenolpyruvate synthase (PEPS)

PEPS activity was determined at 70°C using a discontinuous assay according to Eyzaguirre et al. [55]. The standard assay (total volume 25–50 μl) was performed in 100 mM Tris/HCl, pH 7.0 (at 70°C), in the presence of 30 mM β-mercaptoethanol, 0.1–10 mM pyruvate, 0.1–10 mM ATP, 10 mM MgCl_2_ and 20 μg of enzyme. The amount of PEP formed by PEPS activity after 60–300 s was determined at room temperature in 0.5 ml 100 mM Tris/ HCl, pH 7.0, 20 mM MgCl_2_, 1 mM ADP and 0.8 mM NADH by calculating the decrease in absorption at 366 nm using 10 U lactate dehydrogenase (LDH, rabbit muscle) and 5 U PK (rabbit muscle) as auxiliary enzymes.

Metabolomics experiments were conducted as previously described [15],[50].

## Supporting information

S1 Table System of ordinary differential equations. (PDF)

S2 Table Mathematical description of each reaction of the mathematical model. (PDF)

S3 Table Identification of each reaction of the mathematical model. (PDF)

S4 Table Estimated values/ratios for NADPH/NADP, ATP/ADP, n, P_i_, alpha and volume factor. (PDF)

S1 Fig The influence of co-factors on the metabolites’ steady state. (PDF)

S2 Fig The influence of temperature on the overall system. (PDF)

S3 Fig Model validation. (PDF)

S4 Fig The effect of branch deletion on the ED pathway. (PDF)

S1 Analysis Sensitivity analysis. (PDF)

S2 Analysis Analysis of the feedforward mechanism. (PDF)

S3 Analysis Identifiability analysis. (PDF)

S1 Text Relative standard deviation. (PDF)

S1 Dataset Description of metabolites (name, initial concentration, half-life and sink). (PDF)

S2 Dataset Parameter values (V_max_, K_m_, K_s_, K_i_). (PDF)

## Abbreviations

*S*.*solfataricus*: *Sulfolobus solfataricus*
CCM: central carbohydrate metabolism
Pyr: pyruvate
TCA cycle: tricarboxylic acid cycle
Glc: D-glucose
Gly: glycerate
ED pathway: modified Entner-Doudoroff pathway
npED: non-phosphorylative branch
spED: semi-phosphorylative branch
ATP: adenosine-5'-triphosphate
V_GK_: rate of glycerate kinase
V_KDGKi_: rate of 2-keto-3-deoxy-D-gluconate kinase
V_KD(P)GA_: rate of 2-keto-3-deoxy(-6-phospho) gluconate aldolase
ODE: Ordinary Differential Equations
D-Gat: D-gluconate
KDG: 2-keto-3-deoxygluconate
ADP: adenosine-5'-diphosphate
1,3-BPG: 1,3-bisphosphoglycerate
2-PG: 2-phosphoglycerate
3-PG: 3-phosphoglycerate
V_IPGAM_: rate of phosphoglycerate mutase
KDPG: 2-keto-3-deoxy-6-phosphogluconate
GAP: glyceraldehyde 3-phosphate
G1P: glucose 1-phosphate
PEP: phosphoenolpyruvate
GA: glyceraldehyde
V_Up_: glucose uptake rate
V_GDH_: rate of glucose-1-dehydrogenase
V_GAD_: rate of gluconate dehydratase
V_GAPN_: rate of non-phosphorylating GAPDH
V_PGK_: rate of phosphoglycerate kinase
V_GAPDH_: rate of glyceraldehyde-3-phosphate dehydrogenase
v_ENO_: rate of enolase
V_PK_: rate of pyruvate kinase
V_PEPS_: rate of phosphoenolpyruvate synthetase
V_GAOR_: rate of glyceraldehyde:ferredoxin oxidoreductase
CE: crude extract
Fd_Ox/red_: ferredoxin oxidized/reduced
