# Supplementary material for: Systems biology of the modified branched Entner-Doudoroff pathway in Sulfolobus solfataricus
Source: PLoS One. 2017 Jul 10;12(7):e0180331. doi: 10.1371/journal.pone.0180331 (PMC5503249; doi:10.1371/journal.pone.0180331)
Supplement: S1 Table — (PDF) [file pone.0180331.s001.pdf]

Supporting Information 1

Table S1: System of ordinary differential equations

$$\frac{dGlc}{dt} = V_{Up} - V_{GDH} \quad (S1)$$

$$\frac{dGAT}{dt} = V_{GDH} - V_{GAD} \quad (S2)$$

$$\frac{dKDG}{dt} = V_{GAD} - V_{KDGKI} - V_{KDPGA1} \quad (S3)$$

$$\frac{dKDPG}{dt} = V_{KDGKI} - V_{KDPGA2} \quad (S4)$$

$$\frac{dGAP}{dt} = V_{KDPGA2} + V_{GADPH} - V_{GAPN} - V_{degGAP} - V_{sinkGAP} \quad (S5)$$

$$\frac{dBPG}{dt} = V_{PGK} - V_{GADPH} - V_{degBPG} \quad (S6)$$

$$\frac{d3PG}{dt} = V_{GAPN} - V_{PGK} - V_{IPGAM} \quad (S7)$$

$$\frac{d2PG}{dt} = V_{IPGAM} + V_{GK} - V_{Eno} \quad (S8)$$

$$\frac{dPEP}{dt} = V_{Eno} - V_{PK} + V_{PEPS} - V_{degPEP} \quad (S9)$$

$$\frac{dPyr}{dt} = V_{PK} - V_{PEPS} + V_{KDPGA1} + V_{KDPGA2} - V_{sinkPyr} \quad (S10)$$

$$\frac{dGA}{dt} = V_{KDPGA1} - V_{GAOR} \quad (S11)$$

$$\frac{dGly}{dt} = V_{GAOR} - V_{GK} \quad (S12)$$
