# Supplementary material for: Systems biology of the modified branched Entner-Doudoroff pathway in Sulfolobus solfataricus
Source: PLoS One. 2017 Jul 10;12(7):e0180331. doi: 10.1371/journal.pone.0180331 (PMC5503249; doi:10.1371/journal.pone.0180331)
Supplement: S2 Table — (PDF) [file pone.0180331.s002.pdf]

Supporting Information 2: Mathematical description of each reaction of the mathematical model

Table S2: Rate equations for each reaction

Reaction			
**ID**	**Biochemical Description**	**Equation**	**Generic description**
$\mathbf{v_{Up}}$	$\rightarrow Glc$	Vm_{vUp}^{Glc}	Uptake rate
$\mathbf{v_{GDH}}$	$Glc + NAD(P)^{+} \rightarrow DGat + NAD(P)H^{+}$	$\frac{Vm_{vGDH}^{Glc}\ \mathrm{Glc}}{(Km_{vGDH}^{Glc} + \mathrm{Glc})}$	Irreversible Michaelis Menten (MM)
$\mathbf{v_{GAD}}$	$DGat \rightarrow KDG + H_2O$	$\frac{Vm_{vGAD}^{DGat}\ \mathrm{DGAT}[t]}{(\ Km_{vGAD}^{DGat} + \mathrm{DGAT}[t])}$	Irreversible MM
$\mathbf{v_{KDGKi}}$	$KDG + ATP \rightarrow KDPG + ADP$	$\frac{Vm_{vKDGKi}^{KDG}\ KDG[t]\ ATP}{Km_{vKDGKi}^{KDG}\ Km_{vKDGKi}^{ATP} + KDG[t]\ Km_{vKDGKi}^{ATP} + ATP\ Km_{vKDGKi}^{KDG} + KDG[t]ATP}$	Irreversible MM producing ADP
$\mathbf{v_{KDPGA1}}$	$KDG \leftrightarrow GA + Pyr$	$\frac{Vm_{\mathrm{vKDPGA1}}^{\mathrm{KDG}}\mathrm{KDG[t]}}{Km_{\mathrm{vKDPGA1}}^{\mathrm{KDG}} + \mathrm{KDG}[t]} - \frac{Vm_{\mathrm{vKDPGA1}}^{\mathrm{GA}}\mathrm{Pyr}[t]\mathrm{GA}[t]}{Km_{\mathrm{vKDPGA1}}^{\mathrm{Pyr}}\mathrm{GA}[t] + Km_{\mathrm{vKDPGA1}}^{\mathrm{GA}}\mathrm{Pyr}[t] + Km_{\mathrm{vKDPGA1}}^{\mathrm{Pyr}}\ Km_{\mathrm{vKDPGA1}}^{\mathrm{GA}} + \mathrm{Pyr}[t]\mathrm{GA}[t]}$	Reversible MM with 1 substrate and 2 products
$\mathbf{v_{KDPGA2}}$	$KDPG \leftrightarrow GAP + Pyr$	$\frac{Vm_{\mathrm{vKDPGA2}}^{\mathrm{KDPG}}\mathrm{KDPG[t]}}{Km_{\mathrm{vKDPGA2}}^{\mathrm{KDPG}} + \mathrm{KDPG}[t]} - \frac{Vm_{\mathrm{vKDPGA2}}^{\mathrm{GAP}}\mathrm{Pyr}[t]\mathrm{GAP}[t]}{Km_{\mathrm{vKDPGA2}}^{\mathrm{Pyr}}\mathrm{GAP}[t] + Km_{\mathrm{vKDPGA2}}^{\mathrm{GAP}}\mathrm{Pyr}[t] + Km_{\mathrm{vKDPGA2}}^{\mathrm{Pyr}}Km_{\mathrm{vKDPGA2}}^{\mathrm{GAP}} + \mathrm{Pyr}[t]\mathrm{GAP}[t]}$	Reversible MM with 1 substrate and 2 products
$\mathbf{v_{GAPDH}}$	$1{,}3BPG + NAD(P)H \leftrightarrow GAP + Pi + NAD(P)^{+}$	$\frac{\left(Vm_{\mathrm{vGAPDH}}^{\mathrm{BPG}}\ \frac{BPG[t]NADPH}{Km_{\mathrm{vGAPDH}}^{\mathrm{BPG}}\ Km_{\mathrm{vGAPDH}}^{\mathrm{NADPH}}} - Vm_{\mathrm{vGAPDH}}^{\mathrm{GAP}}\frac{GAP[t]NADP\ Pi}{Km_{\mathrm{vGAPDH}}^{\mathrm{GAP}}\ Km_{\mathrm{vGAPDH}}^{\mathrm{NADP}}\ Km_{\mathrm{vGAPDH}}^{\mathrm{Pi}}}\right)\left(\frac{GAP[t]Pi}{Km_{\mathrm{vGAPDH}}^{\mathrm{GAP}}Km_{\mathrm{vGAPDH}}^{\mathrm{Pi}}} + \frac{BPG[t]}{Km_{\mathrm{vGAPDH}}^{\mathrm{BPG}}}\right)^{n-1}}{\left(1 + \frac{NADP}{Km_{\mathrm{vGAPDH}}^{\mathrm{NADP}}} + \frac{NADPH}{Km_{\mathrm{vGAPDH}}^{\mathrm{NADPH}}}\right)\left[1 + \left(\frac{GAP[t]}{Km_{\mathrm{vGAPDH}}^{\mathrm{GAP}}}\right)^{n} + \left(\frac{Pi}{Km_{\mathrm{vGAPDH}}^{\mathrm{Pi}}}\right)^{n} + \left(GAP[t]\frac{Pi}{Km_{\mathrm{vGAPDH}}^{\mathrm{GAP}}Km_{\mathrm{vGAPDH}}^{\mathrm{Pi}}} + \frac{BPG[t]}{Km_{\mathrm{vGAPDH}}^{\mathrm{BPG}}}\right)^{n}\right]}$	Reversible rate equation with 2 substrates and NADPH oxidation
$\mathbf{v_{GAPN}}$	$GAP + NAD(P)^{+} \rightarrow 3PG + NAD(P)H$	$\frac{Vm_{\mathrm{vGAPN}}^{\mathrm{GAP}}\mathrm{GAP}[t]\mathrm{NADP}}{Km_{\mathrm{vGAPN}}^{\mathrm{NADP}}\mathrm{GAP}[t] + Km_{\mathrm{vGAPN}}^{\mathrm{GAP}}\mathrm{NADP} + Km_{\mathrm{vGAPN}}^{\mathrm{NADP}}Km_{\mathrm{vGAPN}}^{\mathrm{GAP}} + \mathrm{GAP}[t]\mathrm{NADP}}$	Irreversible MM with NADPH oxidation

$\mathbf{v_{PGK}}$	$3PG + ATP \leftrightarrow 1{,}3BPG + ADP$	$\frac{Vm_{\mathrm{vPGK}}^{\mathrm{PG3}} \frac{ATP\ 3PG[t]}{Km_{\mathrm{vPGK}}^{\mathrm{ATP}} Km_{\mathrm{vPGK}}^{\mathrm{3PG}}} - Vm_{\mathrm{vPGK}}^{\mathrm{BPG}} \frac{ADP\ BPG[t]}{Km_{\mathrm{vPGK}}^{\mathrm{ADP}} Km_{\mathrm{vPGK}}^{\mathrm{BPG}}}}{\left(1 + \frac{ADP}{Ki_{\mathrm{vPGK}}^{\mathrm{ADP}}}\right) \left[1 + \frac{3PG[t]}{Km_{\mathrm{vPGK}}^{\mathrm{3PG}}} \left(1 + \frac{ATP}{Km_{\mathrm{vPGK}}^{\mathrm{ATP}}}\right) + \frac{BPG[t]}{Km_{\mathrm{vPGK}}^{\mathrm{BPG}}} \left(1 + \frac{ADP}{Km_{\mathrm{vPGK}}^{\mathrm{ADP}}}\right)\right]}$	Rate equation with substrate inhibition and ATP production
$\mathbf{v_{IPGAM}}$	$3PG \leftrightarrow 2PG$	$\frac{Vm_{vIPGAM}^{3PG}\ \mathrm{3PG[t]}}{(Km_{vIPGAM}^{3PG} + \mathrm{3PG[t]})} - \frac{Vm_{vIPGAM}^{2PG}\ \mathrm{2PG[t]}}{(Km_{vIPGAM}^{2PG} + \mathrm{2PG[t]})}$	Reversible MM
$\mathbf{v_{ENO}}$	$2PG \leftrightarrow PEP$	$\frac{Vm_{vENO}^{2PG}\ \mathrm{2PG[t]}}{(Km_{vENO}^{2PG} + \mathrm{2PG[t]})} - \frac{Vm_{vENO}^{PEP}\ \mathrm{PEP[t]}}{(Km_{vENO}^{PEP} + \mathrm{PEP[t]})}$	Reversible MM
$\mathbf{v_{GK}}$	$Gly + ATP \rightarrow 2PG + ADP$	$Vm_{\mathrm{vGK}}^{\mathrm{Gly}} \frac{Gly[t]^2}{\left(Ks_{\mathrm{vGK}}^{\mathrm{Gly}} + Gly[t]^2 + \frac{Gly[t]^3}{Ki_{\mathrm{vGK}}^{\mathrm{Gly}}}\right)} \left(1 + \frac{\alpha Gly[t]}{Ki_{\mathrm{vGK}}^{\mathrm{Gly}}}\right)$	MM with substrate inhibition and ADP production
$\mathbf{v_{PK}}$	$PEP + ADP \rightarrow Pyr + ATP$	$\frac{Vm_{vPK}^{PEP}\ PEP[t]\ ADP}{Km_{vPK}^{PEP}\ Km_{vPK}^{ADP} + PEP[t]\ Km_{vPK}^{ADP} + ATP\ Km_{vPK}^{PEP} + PEP[t]ADP}$	Irreversible MM with ADP
$\mathbf{v_{PEPS}}$	$Pyr + ATP + H_2O \rightarrow PEP + AMP + Pi$	$\frac{Vm_{vPEPS}^{Pyr}\ Pyr[t]\ ATP}{Km_{vPEPS}^{Pyr}\ Km_{vPEPS}^{ATP} + PEP[t]\ Km_{vPEPS}^{ATP} + ATP\ Km_{vPEPS}^{Pyr} + Pyr[t]ATP}$	Irreversible MM with ATP phosphorylation
$\mathbf{v_{GAOR}}$	$GA + Fd_{ox} \rightarrow Gly + Fd_{red}$	$\frac{Vm_{vGAOR}^{GA}\ \mathrm{GA[t]}}{(Km_{vGAOR}^{GA} + \mathrm{GA[t]})}$	Irreversible MM
$\mathbf{v_{deg}}$	$S \rightarrow$	$\mathrm{K_{deg}} * [S]$	Degradation rate
