# Supplementary material for: Systems biology of the modified branched Entner-Doudoroff pathway in Sulfolobus solfataricus
Source: PLoS One. 2017 Jul 10;12(7):e0180331. doi: 10.1371/journal.pone.0180331 (PMC5503249; doi:10.1371/journal.pone.0180331)
Supplement: S3 Table — (PDF) [file pone.0180331.s003.pdf]

Supporting information 5: Identification of each reaction of the mathematical model.

Table S5: Identification of each reaction. Rev. stands for reversible.

| Reaction ID | EC Number | Sso ID | Enzyme Name | Reaction | Rev? |
|---|---|---|---|---|---|
| $\mathbf{v_{GDH}}$ | 1.1.1.359 | sso3003 | glucose 1-dehydrogenase | $Glc + NAD(P)^+ \rightarrow DGat + NAD(P)H^+$ | No |
| $\mathbf{v_{GAD}}$ | 4.2.1.140 | sso3198 | gluconate/galactonate dehydratase | $DGat \rightarrow KDG + H_2O$ | No |
| $\mathbf{v_{KDGKi}}$ | 2.7.1.178 | sso3195 | 2-keto-3-deoxy-D-gluconate kinase | $KDG + ATP \rightarrow KDPG + ADP$ | No |
| $\mathbf{v_{KDPGA1}}$ | 4.1.2.55 | sso3197 | 2-keto-3-deoxy gluconate aldolase | $KDG \leftrightarrow GA + Pyr$ | Yes |
| $\mathbf{v_{KDPGA2}}$ | 4.1.2.55 | sso3197 | 2-keto-3-deoxy gluconate aldolase | $KDPG \leftrightarrow GAP + Pyr$ | Yes |
| $\mathbf{v_{GAPDH}}$ | 1.2.1.12<br>1.2.1.13 | sso0528 | phosphorylating glyceraldehyde-3-phosphate dehydrogenase | $1{,}3BPG + NAD(P)H \leftrightarrow GAP + Pi + NAD(P)^+$ | Yes |
| $\mathbf{v_{GAPN}}$ | 1.2.1.9 | sso3194 | Non-phosphorylating glyceraldehyde-3-phosphate dehydrogenase | $GAP + NAD(P)^+ \rightarrow 3PG + NAD(P)H$ | No |
| $\mathbf{v_{PGK}}$ | 2.7.2.3 | sso0527 | phosphoglycerate kinase | $3PG + ATP \leftrightarrow 1{,}3BPG + ADP$ | Yes |
| $\mathbf{v_{IPGAM}}$ | 5.4.2.12 | sso0417 | phosphoglycerate mutase | $3PG \leftrightarrow 2PG$ | Yes |
| $\mathbf{v_{ENO}}$ | 4.2.1.11 | sso0913 | enolase | $2PG \leftrightarrow PEP$ | Yes |
| $\mathbf{v_{GK}}$ | 2.7.1.165 | sso0666 | glycerate kinase | $Gly + ATP \rightarrow 2PG + ADP$ | No |
| $\mathbf{v_{PK}}$ | 2.7.1.40 | sso981 | pyruvate kinase | $PEP + ADP \rightarrow Pyr + ATP$ | No |
| $\mathbf{v_{PEPS}}$ | 2.7.9.2 | sso883 | phosphoenolpyruvate synthase | $Pyr + ATP + H_2O \rightarrow PEP + AMP + Pi$ | No |
| $\mathbf{v_{GAOR}}$ | 1.2.99.2 | sso2636; sso2637; sso2639 | glyceraldehyde:Fd oxidoreductase | $GA + Fd_{ox} \rightarrow Gly + Fd_{red}$ | No |
