# Supplementary material for: Systems biology of the modified branched Entner-Doudoroff pathway in Sulfolobus solfataricus
Source: PLoS One. 2017 Jul 10;12(7):e0180331. doi: 10.1371/journal.pone.0180331 (PMC5503249; doi:10.1371/journal.pone.0180331)
Supplement: S4 Table — (PDF) [file pone.0180331.s004.pdf]

Supporting Information 6

Estimated values/ratios for NADPH/NADP, ATP/ADP, n, P_i, alpha and volume factor.

Table S6: Ratio of NADPH/NADP, ATP/ADP and estimated values for n, P_i, alpha. Estimated volume factor.

**NADPH/NADP**	**25**
**ATP/ADP**	1,25
**n**	1,5
**P_i**	100
**alpha**	1
**Volume Factor**	0,250 g/mL
