# Supplementary material for: Systems biology of the modified branched Entner-Doudoroff pathway in Sulfolobus solfataricus
Source: PLoS One. 2017 Jul 10;12(7):e0180331. doi: 10.1371/journal.pone.0180331 (PMC5503249; doi:10.1371/journal.pone.0180331)
Supplement: S1 Fig — (PDF) [file pone.0180331.s005.pdf]

Supporting Information 7

The influence of co-factors on the metabolites' steady state

We changed the ratios of the co-factors ATP, ADP, NAD(P)H+ in our model and simulations reveal that these changes have no impact on 9 of the 12 steady states and low impact on 3 of the 12 steady states of the ED pathway metabolites. Specifically, we increased and decreased the values for ATP, ADP, NAD(P)H+ in 25% for the cases of 80°C and 70°C, as Fig. S1 and S2 show.

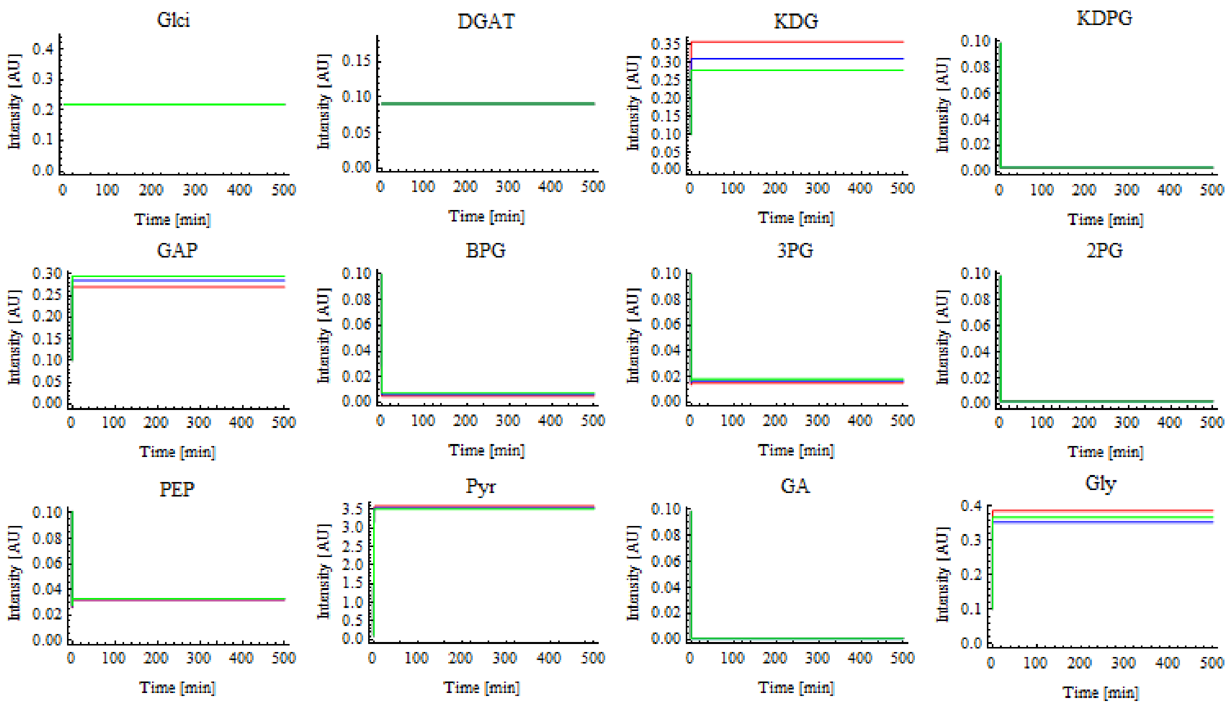

Figure S1. Metabolites time course when ATP, ADP, NAD(P)H+ and NADH vary 25% (increase and decrease), for model parameterized with data at 80°C. Legends: Red: decrease in 25%, Blue: no change, Green: increase in 25%.

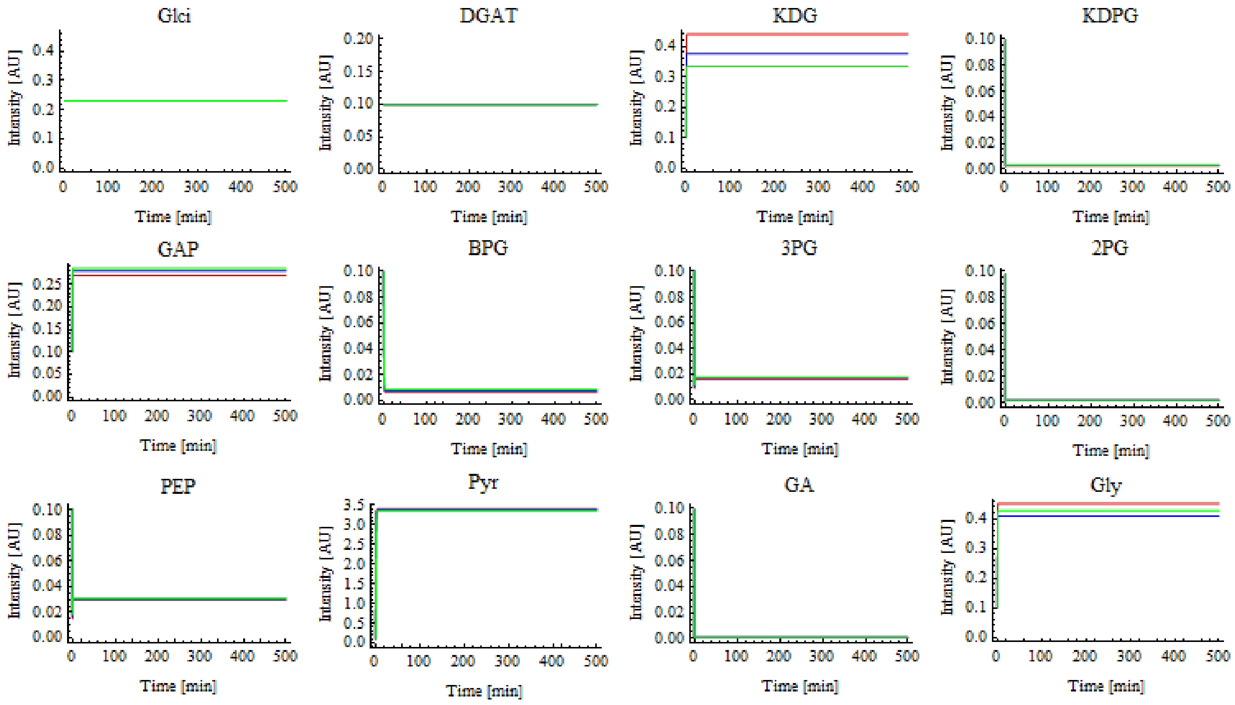

Figure S2. Metabolites time course when ATP, ADP, NAD(P)H+ and NADH vary 25% (increase and decrease), for model parameterized with data at 70°C. Legends: Red: decrease in 25%, Blue: no change, Green: increase in 25%.
