# Supplementary figures and images for: Systems biology of the modified branched Entner-Doudoroff pathway in Sulfolobus solfataricus
Source: PLoS One. 2017 Jul 10;12(7):e0180331. doi: 10.1371/journal.pone.0180331 (PMC5503249; doi:10.1371/journal.pone.0180331)

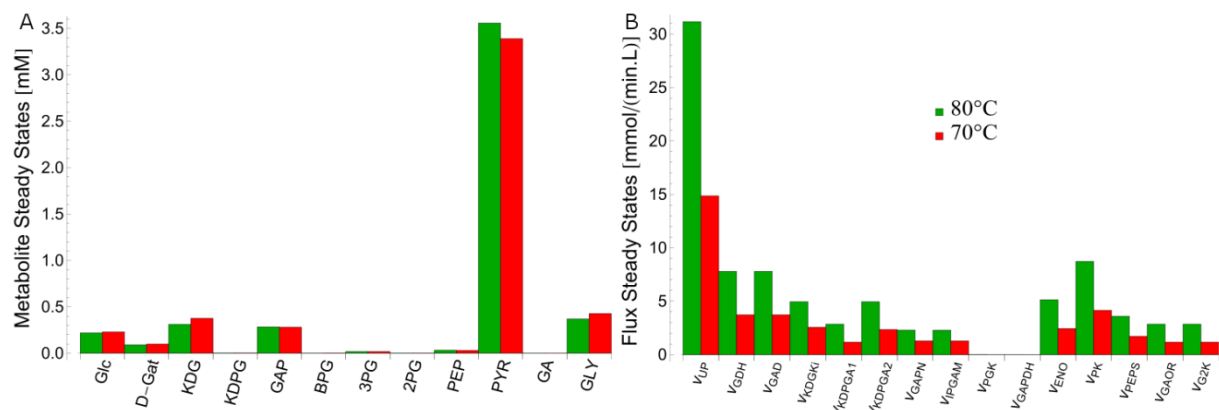

Figure S4. Metabolite A) and flux B) steady states of the ED pathway at 70°C (red) and 80°C (green).

Supplement: S2 Fig — (PDF) [file pone.0180331.s006.pdf]
