# Supplementary material for: Systems biology of the modified branched Entner-Doudoroff pathway in Sulfolobus solfataricus
Source: PLoS One. 2017 Jul 10;12(7):e0180331. doi: 10.1371/journal.pone.0180331 (PMC5503249; doi:10.1371/journal.pone.0180331)
Supplement: S3 Fig — (PDF) [file pone.0180331.s007.pdf]

Supporting Information 9

Model validation

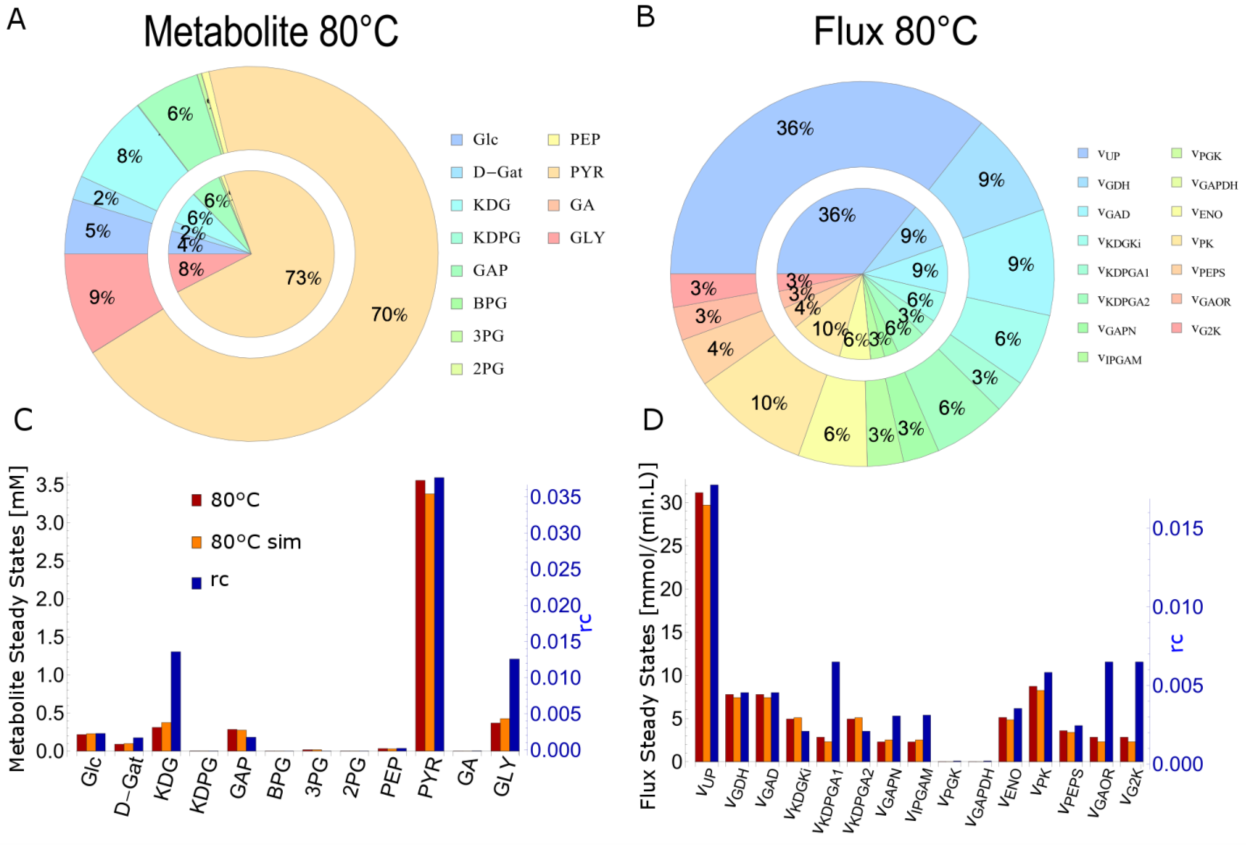

Figure S5: Comparison between $SS_{sim}^{80°C}$and $SS^{80°C}$for metabolite and flux steady states. A-B) Pie Chart. Inner circle: percentual contribution of each metabolite (A) or flux (B) to the respective total at $SS^{80°C}$; Outer circle: percentual contribution of each metabolite (A) or flux (B) to the respective total at $SS_{sim}^{80°C}$ (0% contributions ignored); C-D) Bar Chart comparing the metabolite and flux steady states for $SS_{sim}^{80°C}$ (Orange Bars), $SS^{80°C}$ (dark red bars) and the relative ratio between $SS_{sim}^{80°C}$ and $SS^{80°C}$ (blue bars). The left y-axis refers to the $SS_{sim}^{80°C}$ and $SS^{80°C}$ and the right y-axis (blue) refers to the relative ratio rc. C) Metabolite; D) Flux.
