# Supplementary material for: Systems biology of the modified branched Entner-Doudoroff pathway in Sulfolobus solfataricus
Source: PLoS One. 2017 Jul 10;12(7):e0180331. doi: 10.1371/journal.pone.0180331 (PMC5503249; doi:10.1371/journal.pone.0180331)
Supplement: S4 Fig — (PDF) [file pone.0180331.s008.pdf]

Supporting Information 12

The effect of branch deletion on the ED pathway

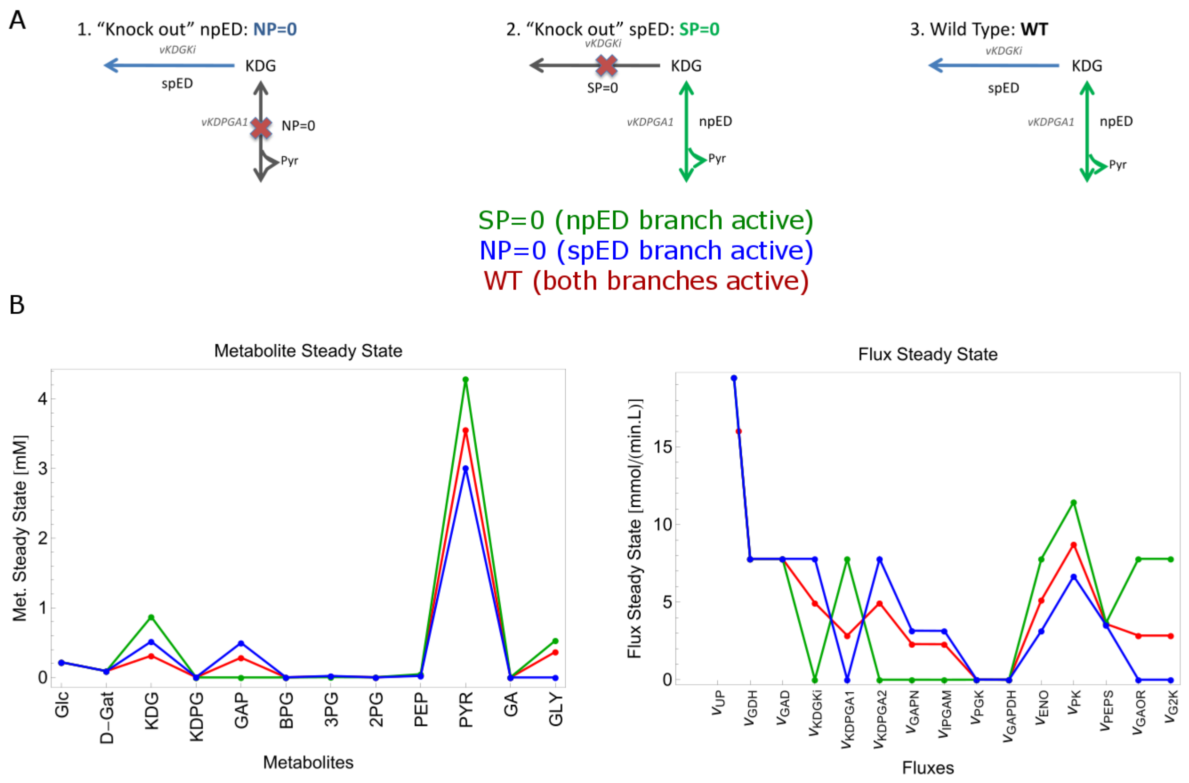

Figure S6. The effect of branch deletion in the ED pathway. A) Schematic representation of the npED branch knock out (NP=0), spED branch knock out (SP=0) and Wild Type (WT). Metabolite B) and Flux C) concentration levels for NP=0 (Blue), SP=0 (Red) and WT (Green).
