# Supplementary material for: Systems biology of the modified branched Entner-Doudoroff pathway in Sulfolobus solfataricus
Source: PLoS One. 2017 Jul 10;12(7):e0180331. doi: 10.1371/journal.pone.0180331 (PMC5503249; doi:10.1371/journal.pone.0180331)
Supplement: S1 Analysis — (PDF) [file pone.0180331.s009.pdf]

Supporting Information 10

Sensitivity analysis

The sensitivity was calculated as:

$$S(O,p) = \frac{\Delta O}{O}\frac{p}{\Delta p} \qquad \text{(S13)}$$

Where O is the output and p is the parameter to perturb. $\Delta O = (\hat{O} - O)$ and $\Delta p = (\hat{p} - p)$. $\hat{O}$ is the output of the model when p is perturbed to $\hat{p}$. For each pair (parameter, metabolite), we show the maximal sensitivity between +20% and -20%, i.e., $Max(S_{-20\%}, S_{+20\%})$. A sensitivity of one indicates that the relative change of the output O is directly proportional to the relative change on the parameter *p*. Generally, a parameter change resulting in -1<S<1 can be termed insensitive, while a sensitivity of *S*>10 indicates a highly sensitive parameter. If *S* is negative, a decrease in p will reflect an increase in *O*, and *vice versa*.

We additionally inspected the sensitivities of Pyr (output) to changes in $Vm_{\mathrm{vUp}}^{\mathrm{Glc}}$ (input), to understand how the input and the output of the ED pathway relate. $Vm_{\mathrm{vUp}}^{\mathrm{Glc}}$ is the V_{max} in the first step of sugar degradation, and Pyr is the end product of the considered ED pathway. Sensitivities of (Pyr, $Vm_{\mathrm{vUp}}^{\mathrm{Glc}}$) are very close to one another at 70°C and 80°C (Table S6).

Table S7: Sensitivities of the pair (Pyr, $Vm_{\mathrm{vUp}}^{\mathrm{Glc}}$).

	**S (Pyr, $Vm_{\mathrm{vUp}}^{\mathrm{Glc}}$)**
**-20% sensitivity at 70°C**	0.97
**+20% sensitivity at 70°C**	0.97
**-20% sensitivity at 80°C**	0.97
**+20% sensitivity at 80°C**	0.96

This indicates that the ratio of perturbations in the input is equal to its effect in the output.
