# Supplementary material for: Systems biology of the modified branched Entner-Doudoroff pathway in Sulfolobus solfataricus
Source: PLoS One. 2017 Jul 10;12(7):e0180331. doi: 10.1371/journal.pone.0180331 (PMC5503249; doi:10.1371/journal.pone.0180331)
Supplement: S2 Analysis — (PDF) [file pone.0180331.s010.pdf]

Supporting Information 13

Analysis of the Feedforward Mechanism.

1. Sensitivity Analysis

We further analyzed the feedforward mechanism acting on reaction v_{GK}. We computed the sensitivity of the metabolites 2-PG, Gly and Pyr when the parameters responsible for the feedforward mechanism ($Ki_{\mathrm{vGK}}^{\mathrm{Gly}}$ and α in Eq. S12.1) change +/- 25%. To calculate the sensitivities, we used the formula present in Eq. S13 (Supporting Information 9). Results indicate that Gly is more vulnerable to these specific changes than 2-PG or Pyr, as Fig. S12.1 shows.

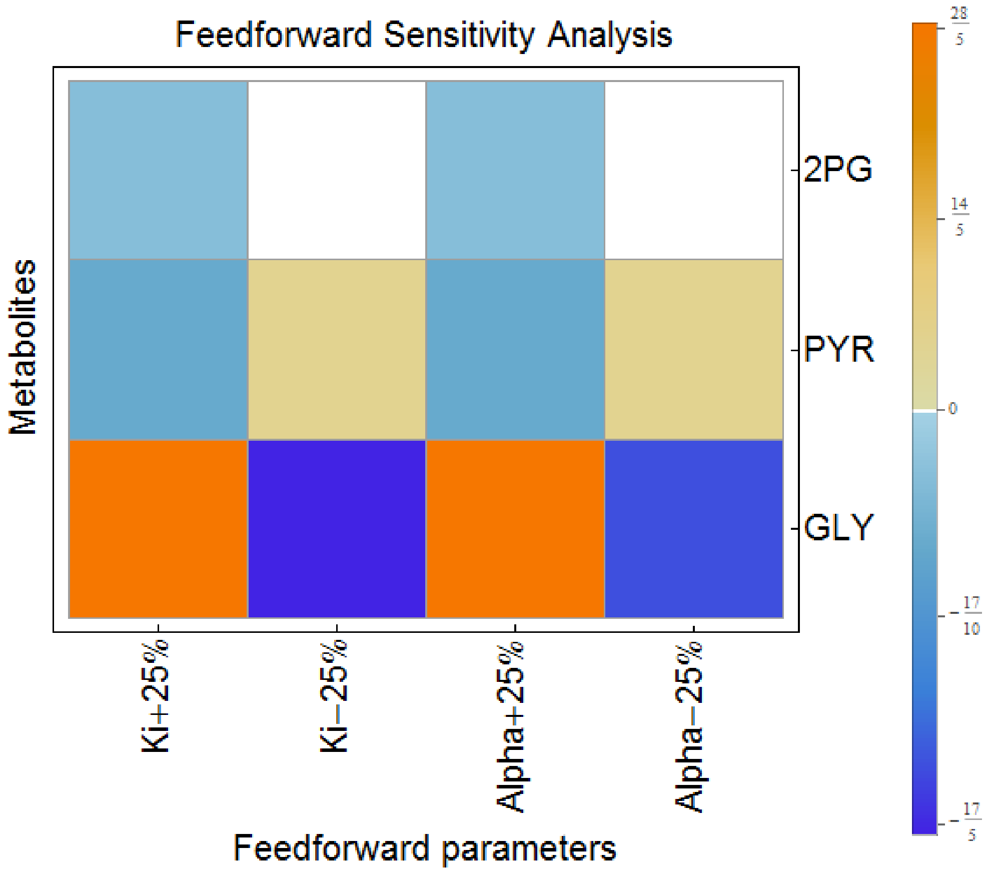

Fig. S6. Sensitivity analysis of the feedforward mechanism. Gly is more sensitive to changes in the parameters $Ki_{\mathrm{vGK}}^{\mathrm{Gly}}$ and α than Pyr or 2-PG. Ki+/-25% represents a change of parameter $Ki_{\mathrm{vGK}}^{\mathrm{Gly}}$ of +/-25% and Alpha+/-25% represents a change of parameter α (Equation S12.1).

2. *In silico* knock-out of the feedforward mechanism

To better understand the role of the feedforward mechanism, we set the parameters that contribute to this regulatory mechanism to zero ($Ki_{\mathrm{vGK}}^{\mathrm{Gly}}$ and α in Eq. S12.1). Equation S12.1 represents the v_{GK} reaction with the feedforward mechanism and equation S12.2 represents this reaction without the feedforward mechanism.

$$Vm_{\mathrm{vGK}}^{\mathrm{Gly}} \frac{Gly[t]^2}{\left(Ks_{\mathrm{vGK}}^{\mathrm{Gly}} + Gly[t]^2 + \frac{Gly[t]^3}{Ki_{\mathrm{vGK}}^{\mathrm{Gly}}}\right)} \left(1 + \frac{\alpha Gly[t]}{Ki_{\mathrm{vGK}}^{\mathrm{Gly}}}\right) \tag{S12.1}$$

$$Vm_{\mathrm{vGK}}^{\mathrm{Gly}} \frac{Gly[t]^2}{\left(Ks_{\mathrm{vGK}}^{\mathrm{Gly}} + Gly[t]^2\right)} \tag{S12.2}$$

We analyzed the effect of this regulatory mechanism at the metabolite level (2-PG, Pyr and Gly) and we observed that the feedforward motif influences the steady state levels of Gly, but not of 2-PG and Pyr (Fig. S12.2).

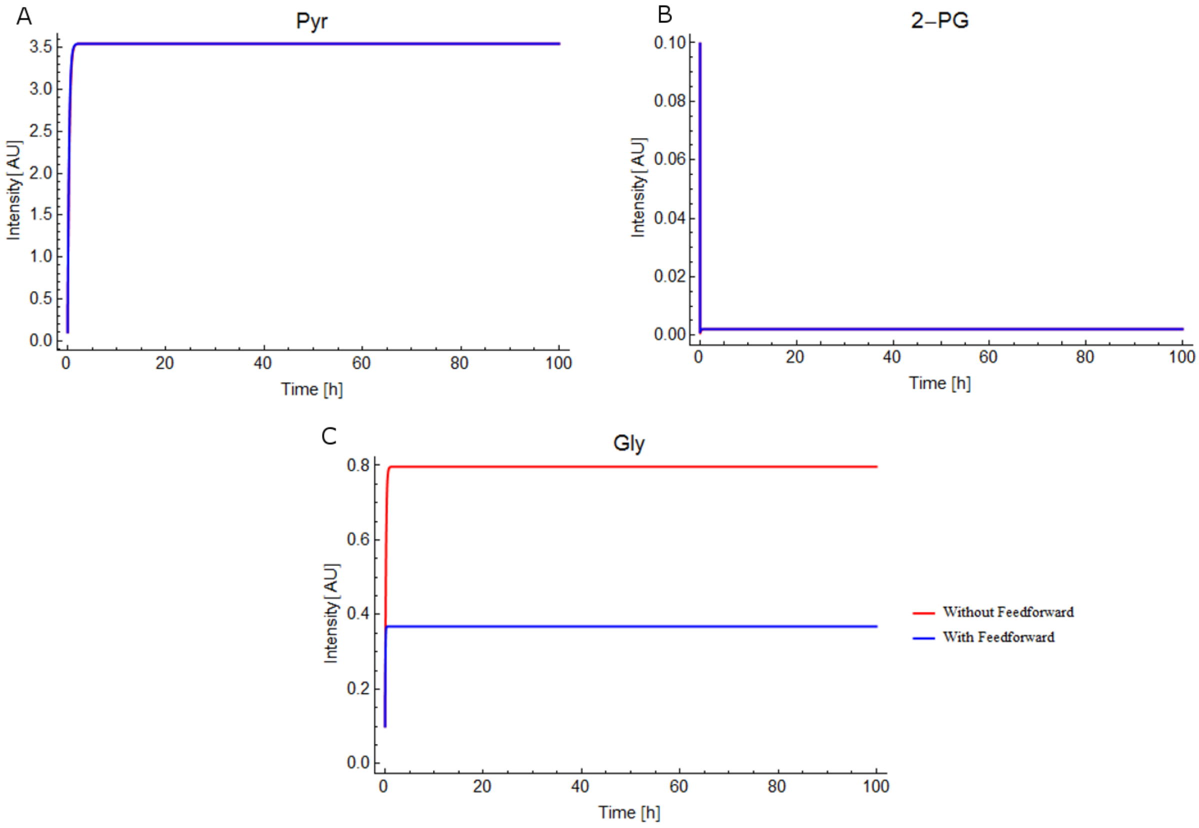

Fig. S7. Time dynamics of the metabolites (A) Pyr, (2) 2-PG and (3) Gly with and without the feedforward mechanism in reaction v_{GK}. This mechanism affects the steady state of Gly, but not of Pyr or 2-PG.

We also analyzed the dependency of v_{GK} in terms of time and Gly concentration. Results clearly show that the feedforward mechanism induces a faster response of v_{GK} not only in its dynamics along time, but also in terms of Gly concentration dependency, as Fig. S12.3 shows.

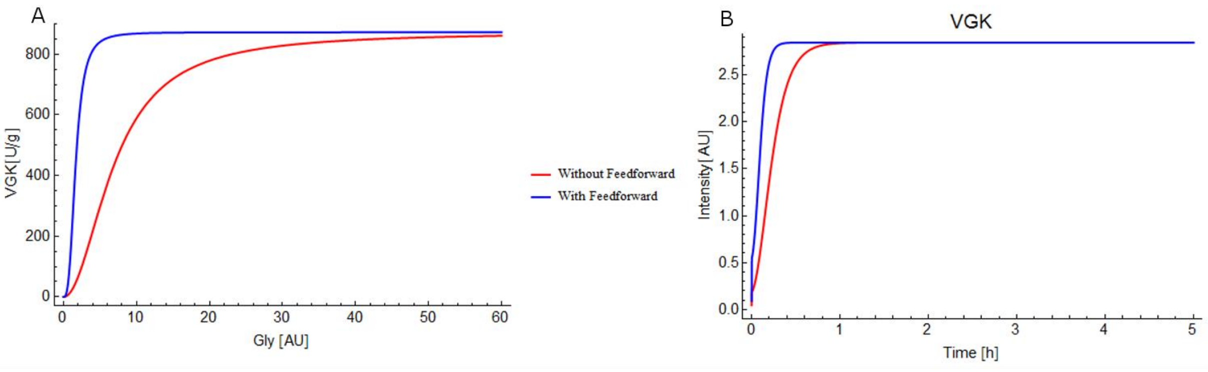

Fig. S8. A) v_{GK} dependency of Gly concentration. B) v_{GK} dynamics in time. With feedforward mechanism (blue) and without (red)
