# Supplementary material for: Systems biology of the modified branched Entner-Doudoroff pathway in Sulfolobus solfataricus
Source: PLoS One. 2017 Jul 10;12(7):e0180331. doi: 10.1371/journal.pone.0180331 (PMC5503249; doi:10.1371/journal.pone.0180331)
Supplement: S3 Analysis — (PDF) [file pone.0180331.s011.pdf]

Supporting Information 14

Identifiability Analysis.

We performed an identifiability analysis to determine how well the parameter $Vm_{\mathrm{vUp}}^{\mathrm{Glc}}$ (at 70°C and 80°C) represents the experimental data. To this end, we varied $Vm_{\mathrm{vUp}}^{\mathrm{Glc}}$ between $0.1 * Vm_{\mathrm{vUp}}^{\mathrm{Glc}}$ and $10 * Vm_{\mathrm{vUp}}^{\mathrm{Glc}}$ and we sampled 10 values from this interval and included the original $Vm_{\mathrm{vUp}}^{\mathrm{Glc}}$ value. We set $Vm_{\mathrm{vUp}}^{\mathrm{Glc}}$ constant to each of these values and we estimated all the other parameters as described in Materials and Methods, Parameter estimation. Fig. S9 illustrates this analysis.

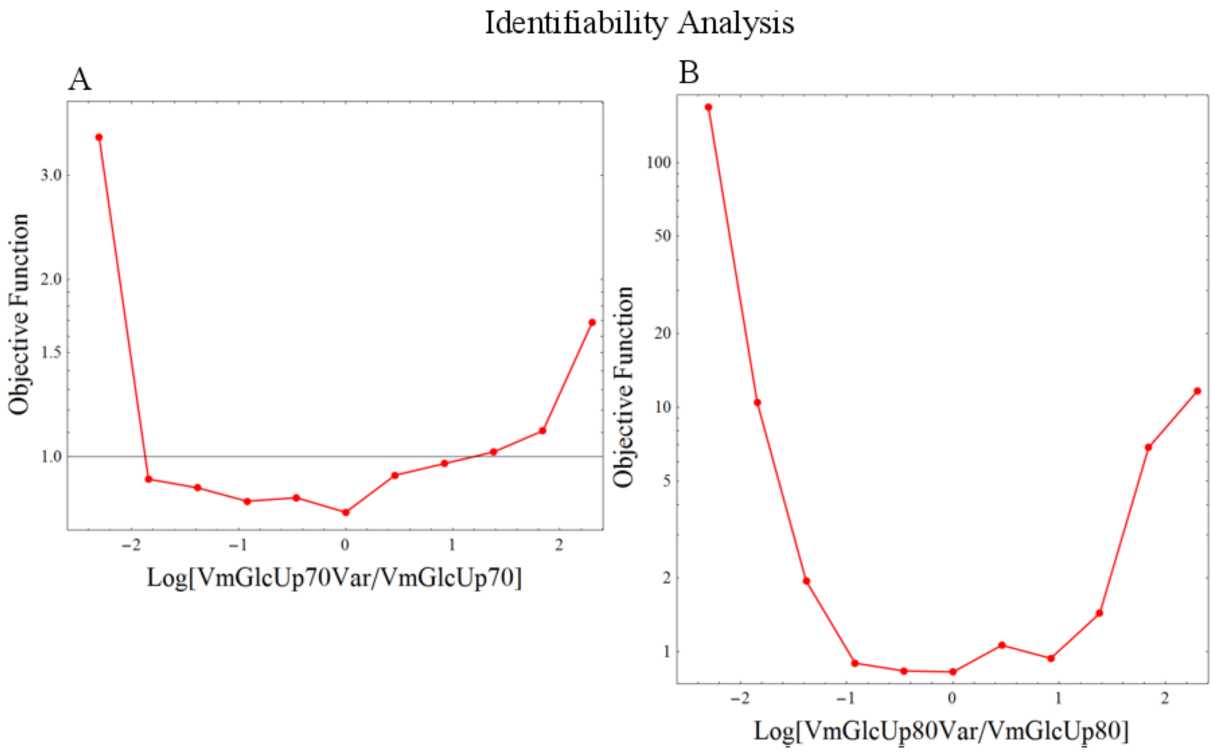

Fig. S9. Identifiability analysis of $Vm_{\mathrm{vUp}}^{\mathrm{Glc}}$ at 70°C (A) and 80°C (B). These results represent the objective function minimization problem used for parameter estimation, when $Vm_{\mathrm{vUp}}^{\mathrm{Glc}}$ varies. The optimal solution presents the lowest objective function. This is, in both cases, the original value of $Vm_{\mathrm{vUp}}^{\mathrm{Glc}}$. These results indicate that this parameter is identifiable both at 70°C and 80°C. VmGlcUp70Var = {1.4856, 4.1596, 6.8337, 9.508, 12.182, 14.8559, 30.9, 60.315, 89.73, 119.14, 148.56}; VmGlcUp80Var = {3.1154, 8.7231, 14.331, 19.939, 25.546, 31.154, 64.8, 126.49, 188.17, 249.86, 311.54}.
