# Supplementary material for: Systems biology of the modified branched Entner-Doudoroff pathway in Sulfolobus solfataricus
Source: PLoS One. 2017 Jul 10;12(7):e0180331. doi: 10.1371/journal.pone.0180331 (PMC5503249; doi:10.1371/journal.pone.0180331)
Supplement: S1 Text — (PDF) [file pone.0180331.s012.pdf]

Supporting Information 11

Relative Standard Deviation:

To measure the relative dispersion of metabolite steady states, we used the relative standard deviation (RSD), with the formula:

$$RSD = \frac{\delta^2}{\overline{ss}} * 100 \qquad \text{(S14)}$$

Where δ^2 is the standard deviation and $\overline{ss}$ is the average of the steady state distribution of each metabolite. RSD is expressed as a percentage of the steady state.
