# Supplementary material for: Systems biology of the modified branched Entner-Doudoroff pathway in Sulfolobus solfataricus
Source: PLoS One. 2017 Jul 10;12(7):e0180331. doi: 10.1371/journal.pone.0180331 (PMC5503249; doi:10.1371/journal.pone.0180331)
Supplement: S1 Dataset — (PDF) [file pone.0180331.s013.pdf]

Supporting Information 3: Description of Metabolites (Name, initial concentration, Half-life and Sink)

Table S3: **Metabolite description**. Names, abbreviations, initial concentrations and degradation rates (half lifes). Half lifes were converted to min^{-1} and allowed to vary +/- 10% around their measured value. *Estimated parameter

Met.	Name	Init Conc [mM]	Half Life [1/min]			Sink
			70 °C	80°C	Par.	$(\boldsymbol{K_{sink}^{Met}})$
**Glc**	glucose (internal)	$\frac{Km_{vGDH}^{Glc}\, Vm_{vUp}^{Glc}}{Vm_{vGDH}^{Glc} - Vm_{vUp}^{Glc}} = 0.22$				
**D-Gat**	D-gluconate	$\left(\frac{\mathrm{Glc}\, Km_{\mathrm{vGAD}}^{\mathrm{DGat}} Vm_{vGDH}^{Glc}}{\mathrm{Glc0}\, Vm_{\mathrm{vGAD}}^{\mathrm{DGat}} + Km_{vGDH}^{Glc} Vm_{\mathrm{vGAD}}^{\mathrm{DGat}} - \mathrm{Glc0}\, Vm_{vGDH}^{Glc}} = 0.09\right)$				
**KDG**	2-keto-3-deoxygluconate	0.1				
**KDPG**	2-keto-3-deoxy-6-phosphogluconate	0.1				
**GAP**	(glyceraldehyde 3-phosphate	0.1	0.056	0.433	K_{deg}^{GAP}	$K_{sink}^{GAP} = 8.99$ *
**3-PG**	3-phosphoglycerate	0.1				
**1,3-BPG**	1,3-bisphospho-glycerate	0.1	1.058	4.027*	K_{deg}^{BPG}	
**2-PG**	2-phosphoglycerate	0.1				
**GA**	glyceraldehyde	0,1				
**Gly**	glycerate	0.1				
**PEP**	phospho-enolpyruvate	0.1	0.008	0.032	K_{deg}^{PEP}	
**Pyr**	pyruvate	0.1				$K_{sink}^{Pyr} = 3.63$ *
