# Supplementary material for: Systems biology of the modified branched Entner-Doudoroff pathway in Sulfolobus solfataricus
Source: PLoS One. 2017 Jul 10;12(7):e0180331. doi: 10.1371/journal.pone.0180331 (PMC5503249; doi:10.1371/journal.pone.0180331)
Supplement: S2 Dataset — (PDF) [file pone.0180331.s014.pdf]

Supporting information 4: Parameter values (V_{max}, K_m, K_s, K_i).

Table S4: Parameter values (V_{max}, K_m, K_s, K_i). * Estimated values for V_{max}. *S. solfataricus* cells were grown at either 70°C or 80°C and enzyme assays were performed at the respective temperature.
The ratio between the averaged enzyme activity for 80°C and 70°C is 2.1.

Reaction ID	Enzyme Activity [U/g CE]			Km, Ks or Ki			
	Parameter Name	70 °C	80°C	Parameter Name	Value [mM]	Temp [°C]	Ref
$\mathbf{v_{Up}}$	Vm_{vUp}^{Glc}	14.86 *	31.15 *				
$\mathbf{v_{GDH}}$	Vm_{vGDH}^{Glc}	96 (+/-6)	250 (+/-5)	Km_{vGDH}^{Glc}	1.50	70	(1)
$\mathbf{v_{GAD}}$	$Vm_{\mathrm{vGAD}}^{\mathrm{DGat}}$	92 (+/-5)	170 (+/-11)	$Km_{\mathrm{vGAD}}^{\mathrm{DGat}}$	0.45	78	(2)
$\mathbf{v_{KDGKi}}$	$Vm_{\mathrm{vKDGKi}}^{\mathrm{KDG}}$	410.66 *	943.3 *	$Km_{\mathrm{vKDGKi}}^{\mathrm{KDG}}$	3.60	70	(3)
				$Km_{\mathrm{vKDGKi}}^{\mathrm{ATP}}$	2.80	70	(3)
$\mathbf{v_{KDPGA1}}$	$Vm_{\mathrm{vKDPGA1}}^{\mathrm{KDG}}$	323.74 *	952.39 *	$Km_{\mathrm{vKDPGA1}}^{\mathrm{KDG}}$	25.70	70	(3)
	$Vm_{\mathrm{vKDPGA1}}^{\mathrm{GA}}$	55 (+/-3)	120 (+/-4)	$Km_{\mathrm{vKDPGA1}}^{\mathrm{Pyr}}$	1.00	70	(4)
	$Vm_{\mathrm{vKDPGA1}}^{Pyr}$	55 (+/-3)	120 (+/-4)	$Km_{\mathrm{vKDPGA1}}^{\mathrm{GA}}$	3.90	70	(4)
$\mathbf{v_{KDPGA2}}$	$Vm_{\mathrm{vKDPGA2}}^{\mathrm{KDPG}}$	413.76 *	908.77 *	$Km_{\mathrm{vKDPGA2}}^{\mathrm{KDPG}}$	0.10	70	(1)
	$Vm_{\mathrm{vKDPGA2}}^{\mathrm{GAP}}$	41 (+/-4)	120 (+/-17)	$Km_{\mathrm{vKDPGA2}}^{\mathrm{Pyr}}$	1.00	70	(4)
	$Vm_{\mathrm{vKDPGA2}}^{Pyr}$	41 (+/-4)	120 (+/-17)	$Km_{\mathrm{vKDPGA2}}^{\mathrm{GAP}}$	3.90	70	(4)
$\mathbf{v_{GAPDH}}$	$Vm_{\mathrm{vGAPDH}}^{\mathrm{BPG}}$	117.15 *	933.91 *	$Km_{\mathrm{vGAPDH}}^{\mathrm{BPG}}$	0.0004	70	(5)
	$Vm_{\mathrm{vGAPDH}}^{\mathrm{GAP}}$	43 (+/-4)	87 (+/-9)	$Km_{\mathrm{vGAPDH}}^{\mathrm{NADPH}}$	0.07	70	(5)
				$Km_{\mathrm{vGAPDH}}^{\mathrm{GAP}}$	0.84	70	(5)
				$Km_{\mathrm{vGAPDH}}^{\mathrm{NADP}}$	0.27	70	(5)
				$Km_{\mathrm{vGAPDH}}^{\mathrm{Pi}}$	408.5	70	(5)
				$Km_{\mathrm{vGAPN}}^{\mathrm{GAP}}$	0.5	70	(6)
$\mathbf{v_{GAPN}}$	$Vm_{\mathrm{vGAPN}}^{\mathrm{GAP}}$	21 (+/-1)	36 (+/-1)	$Km_{\mathrm{vGAPN}}^{\mathrm{NADP}}$	0.09	70	(6)
				$Km_{\mathrm{vPGK}}^{\mathrm{PG3}}$	0.54	70	(5)
$\mathbf{v_{PGK}}$	$Vm_{\mathrm{vPGK}}^{\mathrm{PG3}}$	30 (+/-1)	110 (+/-1)	$Km_{\mathrm{vPGK}}^{\mathrm{ATP}}$	9.70	70	(5)
	$Vm_{\mathrm{vPGK}}^{\mathrm{BPG}}$	341.81 *	217.4 *	$Km_{\mathrm{vPGK}}^{\mathrm{BPG}}$	5.59	70	(5)
				$Ki_{\mathrm{vPGK}}^{\mathrm{ADP}}$	1.14	70	(5)
				$Km_{\mathrm{vPGK}}^{\mathrm{ADP}}$	0.09	70	(5)
				$Km_{\mathrm{vIPGAM}}^{\mathrm{PG3}}$	0.10	70	(5)
	$Vm_{\mathrm{vIPGAM}}^{\mathrm{PG3}}$	93	75	$Km_{\mathrm{vIPGAM}}^{\mathrm{PG2}}$	0.15	70	(7)

**v_{IPGAM}**		(+/-2)	(+/-4)				
	Vm^{PG2}_{vIPGAM}	609.81 *	169.76 *	Km^{PG2}_{vENO}	0.09	70	(7)
**v_{ENO}**	Vm^{PG2}_{vENO}	1000 *	937.96 *	Km^{PEP}_{vENO}	0.42	70	(7)
	Vm^{PEP}_{vENO}	199.40 *	10.00 *	Km^{Gly}_{vGK}	48	70	(8)
**v_{GK}**	Vm^{Gly}_{vGK}	236.53 *	872.35 *	Ki^{Gly}_{vGK}	0.1	70	(8)
				Km^{PEP}_{vPK}	0.09	70	(7)
**v_{PK}**	Vm^{PEP}_{vPK}	70 (+/-4)	144 *	Km^{ADP}_{vPK}	0.07	70	(7)
				Km^{Pyr}_{vPEPS}	0.46	70	(7)
**v_{PEPS}**	Vm^{Pyr}_{vPEPS}	12.45 *	26.13 *	Km^{ATP}_{vPEPS}	0.61	70	(7)
**v_{GAOR}**	Vm^{GA}_{vGAOR}	474.72 *	959.63 *	Km^{GA}_{vGAOR}	0.09	80	(9)
**Average of measured and estimated enzyme activity**		48	101				
**Average of measured enzyme activity**		64	154				

1. Lamble HJ, Heyer NI, Bull SD, Hough DW, Danson MJ. Metabolic pathway promiscuity in the archaeon Sulfolobus solfataricus revealed by studies on glucose dehydrogenase and 2-keto-3-deoxygluconate aldolase. The Journal of biological chemistry. 2003;278(36):34066-72.
2. Kim S, Lee SB. Identification and characterization of Sulfolobus solfataricus D-gluconate dehydratase: a key enzyme in the non-phosphorylated Entner-Doudoroff pathway. The Biochemical journal. 2005;387(Pt 1):271-80.
3. Lamble HJ, Theodossis A, Milburn CC, Taylor GL, Bull SD, Hough DW, et al. Promiscuity in the part-phosphorylative Entner-Doudoroff pathway of the archaeon Sulfolobus solfataricus. FEBS letters. 2005;579(30):6865-9.
4. Buchanan CL, Connaris H, Danson MJ, Reeve CD, Hough DW. An extremely thermostable aldolase from Sulfolobus solfataricus with specificity for non-phosphorylated substrates. The Biochemical journal. 1999;343 Pt 3:563-70.
5. Kouril T, Esser D, Kort J, Westerhoff HV, Siebers B, Snoep JL. Intermediate instability at high temperature leads to low pathway efficiency for an in vitro reconstituted system of gluconeogenesis in Sulfolobus solfataricus. The FEBS journal. 2013;280(18):4666-80.
6. Ettema TJ, Ahmed H, Geerling AC, van der Oost J, Siebers B. The non-phosphorylating glyceraldehyde-3-phosphate dehydrogenase (GAPN) of Sulfolobus solfataricus: a key-enzyme of the semi-phosphorylative branch of the Entner-Doudoroff pathway. Extremophiles : life under extreme conditions. 2008;12(1):75-88.
7. Haferkamp P. Biochemical studies of enzymes involved in glycolysis of the thermoacidophilic crenarchaeon Sulfolobus solfataricus. Essen: Universität Duisburg - Essen; 2011.

8. Kouril T, Wieloch P, Reimann J, Wagner M, Zaparty M, Albers SV, et al. Unraveling the function of the two Entner-Doudoroff branches in the thermoacidophilic Crenarchaeon Sulfolobus solfataricus P2. The FEBS journal. 2013;280(4):1126-38.
9. Kardinahl S, Schmidt CL, Hansen T, Anemuller S, Petersen A, Schafer G. The strict molybdate-dependence of glucose-degradation by the thermoacidophile Sulfolobus acidocaldarius reveals the first crenarchaeotic molybdenum containing enzyme--an aldehyde oxidoreductase. European journal of biochemistry / FEBS. 1999;260(2):540-8.
